# The unique interplay of mitochondrial oxidative phosphorylation (OXPHOS) and immunity and its potential implication for the sex‐ and age‐related morbidity of severe COVID‐19 patients

**DOI:** 10.1002/mco2.371

**Published:** 2023-09-23

**Authors:** Yinchuan Li, Lei Li, Guanghao Wu, Gangcai Xie, Lirong Yi, Jie Zhu, ShiYu Liang, Ya‐ru Huang, Juan Chen, Shaoyang Ji, Fei Sun, Rui‐tian Liu

**Affiliations:** ^1^ Institute of Reproductive Medicine Medical School of Nantong University Nantong Jiangsu P. R. China; ^2^ National Clinical Research Center for Obstetric & Gynecologic Diseases Department of Obstetrics and Gynecology Peking Union Medical College Hospital Chinese Academy of Medical Sciences & Peking Union Medical College Beijing P. R. China; ^3^ School of Materials Science and Engineering Beijing Institute of Technology Beijing P. R. China; ^4^ National Key Laboratory of Biochemical Engineering Institute of Process Engineering Chinese Academy of Sciences Beijing P. R. China; ^5^ University of Chinese Academy of Sciences Beijing P. R. China

**Keywords:** androgen, castration, COVID‐19, immunity, oxidative phosphorylation, sex dimorphism

## Abstract

Aged male patients are more vulnerable to severe or critical symptoms of COVID‐19, but the underlying mechanism remains elusive. In this study, we analyzed previously published scRNA‐seq data from a large cohort of COVID‐19 patients, castrated and regenerated mice, and bulk RNA‐seq of a RNAi library of 400 genes, and revealed that both immunity and OXPHOS displayed cell‐type‐, sex‐, and age‐related variation in the severe or critical COVID‐19 patients during disease progression, with a more prominent increase in immunity and decrease in OXPHOS in myeloid cells in the males relative to the females (60–69 years old). Male severe or critical patients above 70 years old were an exception in that the compromised negative correlation between OXPHOS and immunity in these patients was associated with its disordered transcriptional regulation. Finally, the expression levels of OXPHOS and androgens were revealed to be positively correlated, and the responses of macrophages to android fluctuation were more striking than other types of detected immune cells in the castrated mice model. Therefore, the interplay of OXPHOS and immunity displayed a cell‐type‐specific, age‐related, and sex‐biased pattern, and the underlying potential regulatory role of the hormonal milieu should not be neglected.

## Graphical abstract

The immunity and energy metabolism displayed cell‐type‐dependent, age‐related, and sex‐biased characteristics in severe or critical COVID‐19 patients. The immunity of 60‐ to 69‐year‐old male patients was higher than that of the females in several types of immune cells, but it was on the contrary for patients above 70 years old. The underlying correlations of OXPHOS with immunity or androgen were also revealed.

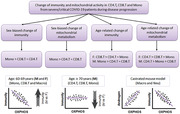

## 1 INTRODUCTION

More and more lines of evidence show that severe or critical syndromes of COVID‐19 are, in many cases, associated with hyperinflammation and cytokine storms.^1^, ^2^, ^3^ Although old age and its co‐morbidities have been regarded as the biggest risk factors for severe and fatal COVID‐19, sex differences are also important factors.^4^, ^5^, ^6^, ^7^ Male patients are more vulnerable to severe or critical symptoms and are at a higher risk of death than females.^8^, ^9^, ^10^, ^11^, ^12^, ^13^, ^14^ Elevated inflammation and cytokine storms have been proposed as contributors to the sex dimorphism in immune responses; in particular, the elevated innate immune reactions seen with some COVID‐19 patients are increasingly attracting attention.^15^, ^16^, ^17^

Sex steroid hormones have been proven to regulate immunity, and androgens especially are able to suppress immune responses.^18^ Low baseline levels of androgens predict a poor prognosis and mortality in COVID‐19 patients.^19^, ^20^, ^21^, ^22^, ^23^ Males are known to have a gradual decline in testosterone levels of around 0.1 nmol/L per year starting from the third decade of life.^24^ Therefore, in addition to sex‐associated immune activities, age‐related immune activity change may be affected by the age‐related decline of androgens in the males, given the important role of androgens in immunity. But the related information is still missing.

Energy metabolism has been regarded as a key factor in regulating immunity in immune cells. For example, the two energetic pathways OXPHOS and glycolysis are reported to be involved in anti‐inflammatory and proinflammatory process, respectively, in macrophages (macro).^25^ Meanwhile, energy partition and utilization are also targets of androgen.^26^ Energy metabolism is, thus, the convergence of both immunity and androgen. But the relation of the energy metabolism and sex dimorphism in immunity, especially in severe or critical COVID‐19 patients, is still unknown.

To decipher the contributing factors of the sex dimorphism in COVID‐19 patients, we compared the immune and metabolic activities of various immune cell populations from severe or critical COVID‐19 patients of both genders in several age groups. To better understand the role of androgens in different populations of immune cells, we analyzed published single‐cell RNA‐sequencing (scRNA‐seq) data for castrated mice to check for the responses of distinct immune cell populations’ immune and metabolic activities. Via comprehensive receiver operating characteristics (ROC) analysis, we were able to obtain the information of cell‐type‐specific, age‐related, and sex‐biased immunity and energy metabolism in severe or critical patients during disease progression (prog) or convalescence (conv). The interplay of mitochondrial OXPHOS and immunity were also illustrated across different immune cell types between both genders, that is, from various mitochondrial metabolic activities, we discovered the OXPHOS subunits may take important roles in responding androgen signals to regulate immune activities in many immune cells, especially in myeloid cells. Additionally, the disorder of the reciprocal correlation of OXPHOS and immunity was revealed in aged male severe or critical COVID‐19 patients (≥70 years old).

## 2 RESULTS

### 2.1 Sex‐dependent, cell‐type‐specific, and age‐related expression profiles of immune activities of severe or critical COVID‐19 patients

**FIGURE 1:**
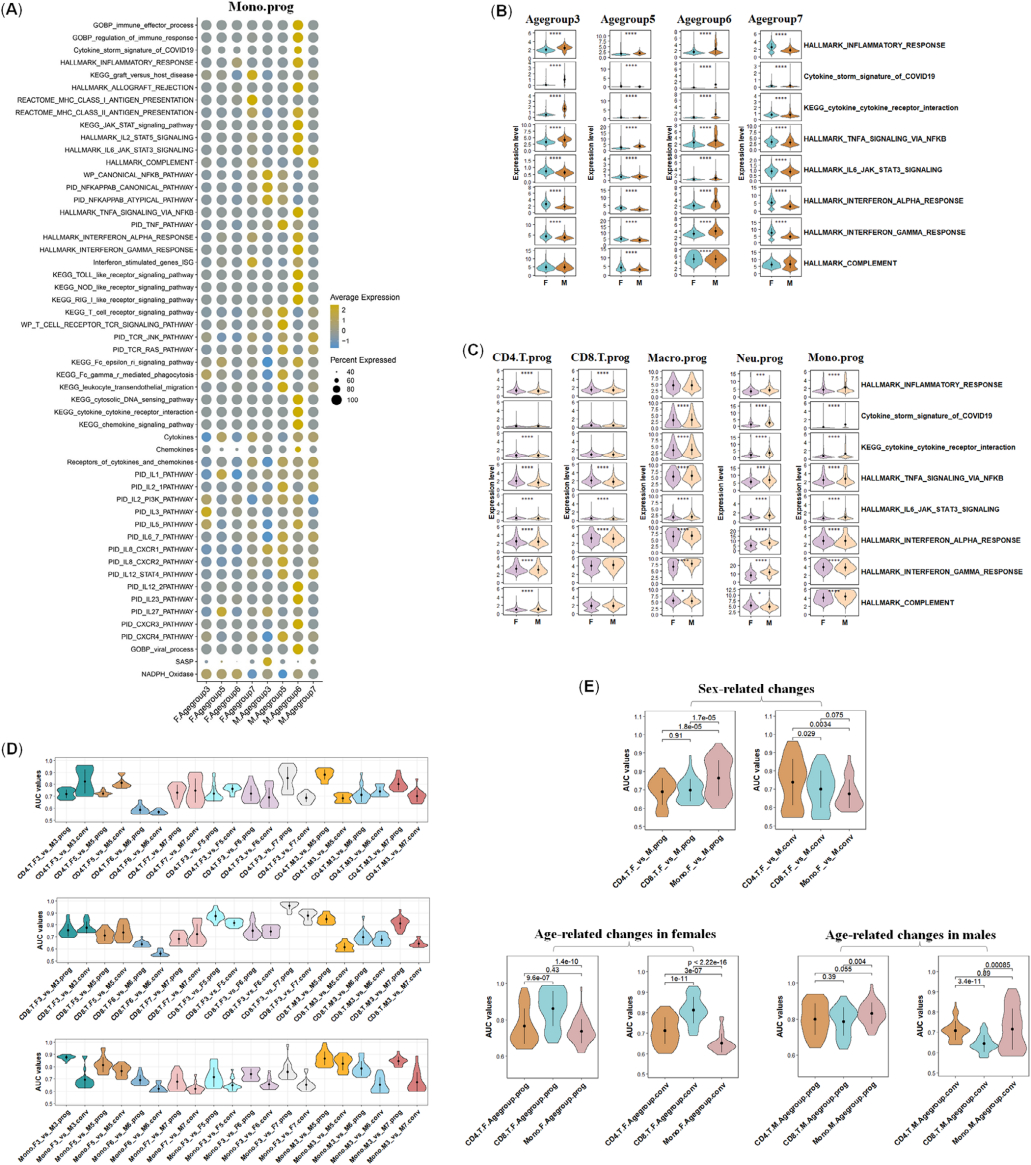
The changes of immune activities in different immune cells across both genders and three comparing age groups from severe or critical COVID‐19 patients. **(A**) Distinct dynamic profiles of 49 immune pathways, gene sets, or signatures in Mono between male and female severe or critical COVID‐19 patients (prog) in four age groups. Age group 3: 30−39 years old, age group 5: 50−59 years old, age group 6: 60−69 years old, age group 7: ≥70 years old. (**B**) Relative expression profiles of eight immune pathways in Mono from four age groups (male or female severe or critical patients (prog). (**C**) Relative expression profiles of eight immune pathways between both genders in five types of immune cells from severe or critical patients (prog). (**D**) Top 20 AUC values of 49 immune pathways, gene sets, or signatures in 10 pairwise comparisons of three types of immune cells from severe or critical COVID‐19 patients (prog or conv). (**E**) Sex‐ or age‐related changes in immune pathways, gene sets, or signatures derived from the accumulation of the top 20 AUC values in each of sex‐related comparing pairs or age‐related comparing pairs in three types of immune cells as those in (**D**). Sex‐related comparing pairs were performed within four age groups between both genders (F3 vs. M3, F5 vs. M5, F6 vs. M6, and F7 vs. M7). Age‐related comparing pairs were the three comparing pairs in females (F3 vs. F5, F3 vs. F6, and F3 vs. F7) and males (M3 vs. M5, M3 vs. M6, and M3 vs. M7), respectively, from severe or critical COVID‐19 patients (prog or conv). *p < 0.05, **p < 0.01, ***p < 0.005, ****p < 0.001, two‐sided Wilcoxon rank‐sum test. All the following settings of the symbol of *p* values were the same. Mean ± SD were labeled.

To monitor the common cell‐type‐specific features in immunity across genders and age groups from COVID‐19 patients, we first checked the immune activities of the scRNA‐seq data from a large cohort of COVID‐19 patients consisting of both genders with ages ranging from 6 to 87 years old (Supporting Information Table S1).^27^ We grouped all the patients into seven age groups according to Pearson's Chi‐squared test between the distribution of mild or moderate and severe or critical patients (df = 6, *p*‐value = 6.528e‐07) (Supporting Information Table S2). The male/female (M/F) ratio of all severe or critical patients in the whole cohort was 1.75 (Supporting Information Table S3). Four age groups (3, 5, 6, 7) were picked for analysis because they included severe or critical patients of both genders (Supporting Information Table S3). The average cell populations of monocytes (mono), CD4^+^ T cells (CD4.T), and CD8^+^ T cells (CD8.T) were much larger than dendritic cells (DC), macrophages (macro), natural killer (NK) cells, and neutrophils (neu) in the four age groups and were, thus, paid more attention in regard to the cell numbers for valid statistical analysis in subgroups divided by disease stages (prog and conv) and genders (Supporting Information Figure S1A, B). In the following analysis, immune cells from severe or critical patients during disease prog were mainly focused. From the overall expression pattern of 49 immune pathways, gene sets, and signatures, we found that mono from severe or critical male patients (prog) displayed higher immune activity levels than those from severe or critical female patients in age groups 5 and 6, a trend more prominent than that in patients (conv) (Figure 1A, Supporting Information Figure S2A). Prior reports of normal people of both genders also revealed similar sex dimorphisms in different types of immune cells when examined by ATAC‐seq, that is, young females usually had higher immune activity levels in their adaptive immune cells, and aged males usually had higher immune activity levels in their innate immune cells.^28^ We then checked the expression levels of eight representative immune pathways, gene sets, and signatures via the Wilcoxon rank‐sum test in the two genders across four age groups of severe or critical patients during disease prog. Mono from severe or critical male patients displayed higher levels of immune activities in age group 6 and lower levels in age group 7 (≥70 years old) than those from severe or critical female patients (Figure 1B). Gene sets of inflammatory response and TNFA signaling via NFKB were higher in age groups 3, 5, 6 in male patients than females. While in age groups 3 and 5, the interferon alpha and beta signaling pathways were lower in male patients than females. This suggested that the factors contributing to the severe or critical manifestations in male COVID‐19 patients of advanced age were different to those in younger male patients, which is supported by prior clinical analysis.^29^ Differences in the overall levels of the eight immune pathways or signatures in innate immune cells (mono, macro, neu, DC, and NK cells) and adaptive cells (CD4.T and CD8.T cells) between both genders of severe or critical patients (prog or conv) (all four age groups) were also calculated via the Wilcoxon rank‐sum test (Figure 1C, Supporting Information Figure S2B, C). Most of the eight pathways or signatures were significantly elevated in the innate immune cells (mono, macro, neu, and DC) of males (prog) compared with those of females. While in CD4.T, CD8.T, and NK cells, most of the eight immune pathways or signatures were largely downregulated in males compared with female severe or critical patients (prog), which is in accordance with early report.^6^ In CD4.T and CD8.T cells from patients during disease conv, most of the eight immune pathways or signatures were still downregulated in males compared with females (Supporting Information Figure S2B).

To more comprehensively compare the alteration in immunity, the ranks of area under curve (AUC) values of 49 immune pathways, gene sets, signatures in mono and two types of adaptive immune cells (CD4.T and CD8.T) were calculated across four age groups in both genders during disease prog and conv, respectively (Figure 1D, Supporting Information Tables [Link], [Link]). The differences in immunity between both genders were more prominent in age groups 3 and 5, and age group 6 had the least differences in these three cell types. In mono, age‐related variation in immunity was more striking in male patients, and in CD8.T.prog, it is more prominent in female patients (prog).

The overall sex‐biased changes of immune activities in four comparing age groups between both genders indicated that Mono.prog changed the most among the three types of immune cells (mono > CD8.T ≈ CD4.T) (Figure 1E). While Mono.conv changed the least between both genders among the three types of immune cells in patients (conv) (CD4.T > CD8.T > mono). In regard to age‐related changes, CD8.T.prog and CD8.T.conv changed the most among the three types of immune cells from female patients during disease prog and conv, respectively. Mono.prog changed the most among the three types of immune cells from male patients (prog) (mono > CD4.T ≈ CD8.T). Therefore, age‐related changes in immune activities of severe or critical COVID‐19 patients (prog) displayed cell‐type‐specific and sex‐biased features (Supporting Information Figures [Link], [Link]).

Collectively, the results strongly suggested that sex‐dependent, cell‐type‐specific, and age‐related variations in immune activities existed in severe or critical COVID‐19 patients.

### 2.2 Age‐related sex bias was remarkable in immunity of severe or critical COVID‐19 patients during disease prog

**FIGURE 2:**
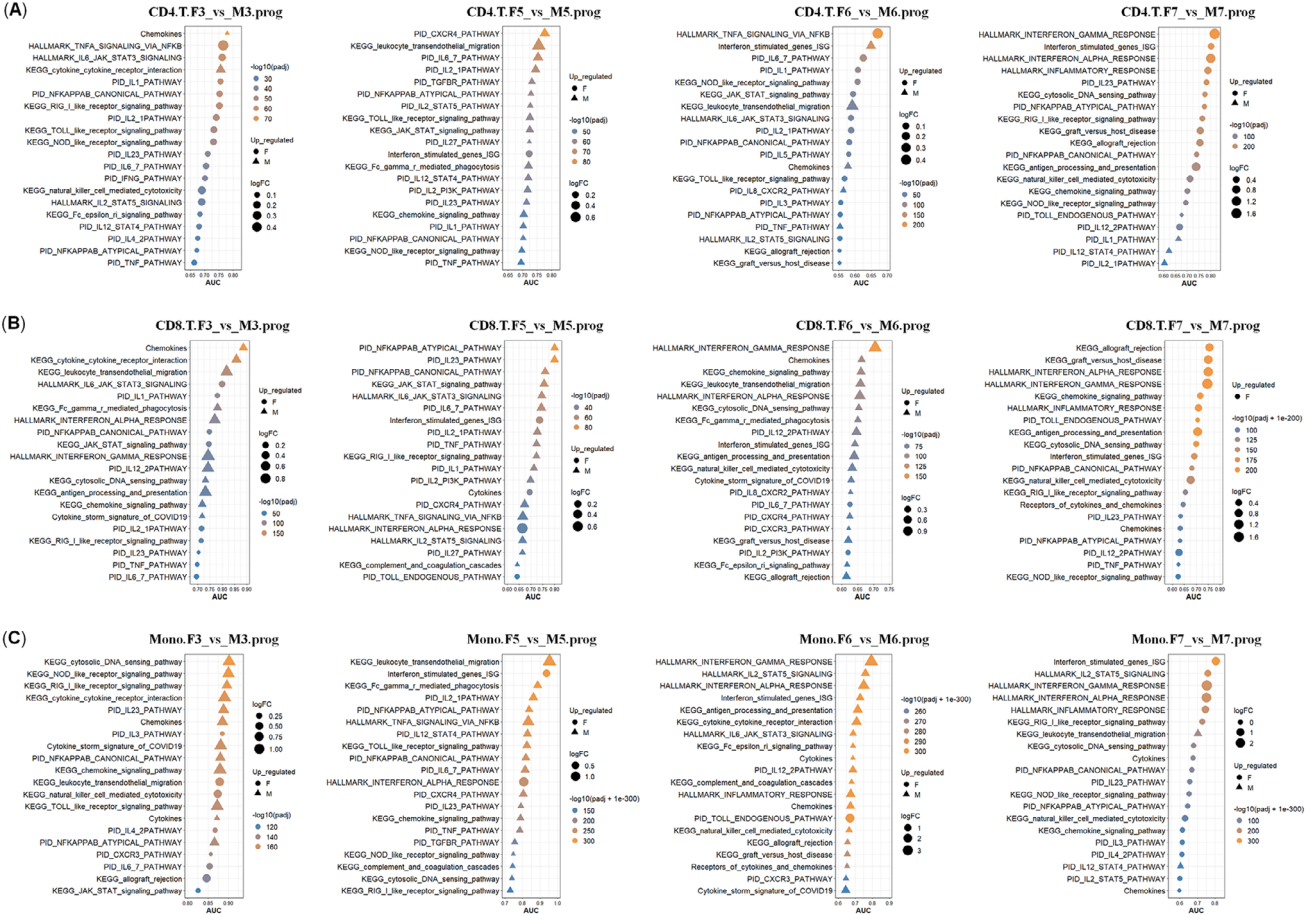
The top 20 changed immune pathways/gene sets/signatures according to the ranks of AUC values between both genders in four age groups from severe/critical COVID‐19 patients (prog) in CD4.T cells (**A**), CD8.T cells (**B**), and in mono cells (**C**).

To obtain detailed information in age‐related sex bias in immunity from severe or critical patients, we profiled the AUC ranks in the three types of immune cells via ROC analysis in 49 immune pathways between both genders in each age group (Supporting Information Tables [Link], [Link]). In CD4.T.prog, the immune activities in female patients from age groups 3, 6, and 7 were higher than those in males (Figure 2A). In CD8.T.prog, the immune activities in male patients from age groups 5 and 6 were higher than those in females (Figure 2B). In Mono.prog, the immune activities in male patients from age groups 3, 5, and 6 were higher than those in females (Figure 2C). In both CD8.T.prog and Mono.prog, the interferon α and γ signaling pathways were on the top ranks in patients from age group 6. Notably, the overall immune activities in male patients from age group 7 were lowered extensively compared to the females in CD4.T.prog, CD8.T.prog, and Mono.prog, suggesting remarkable immune deficiency in these aged male patients.

Therefore, age‐related sex‐bias were very striking in both adaptive and innate immunity in severe or critical COVID‐19 patients (prog), especially in age group 7.

### 2.3 The age‐related, sex‐biased mitochondrial activities, and the negative correlation of OXPHOS with immunity in immune cells from severe or critical COVID‐19 patients

**FIGURE 3:**
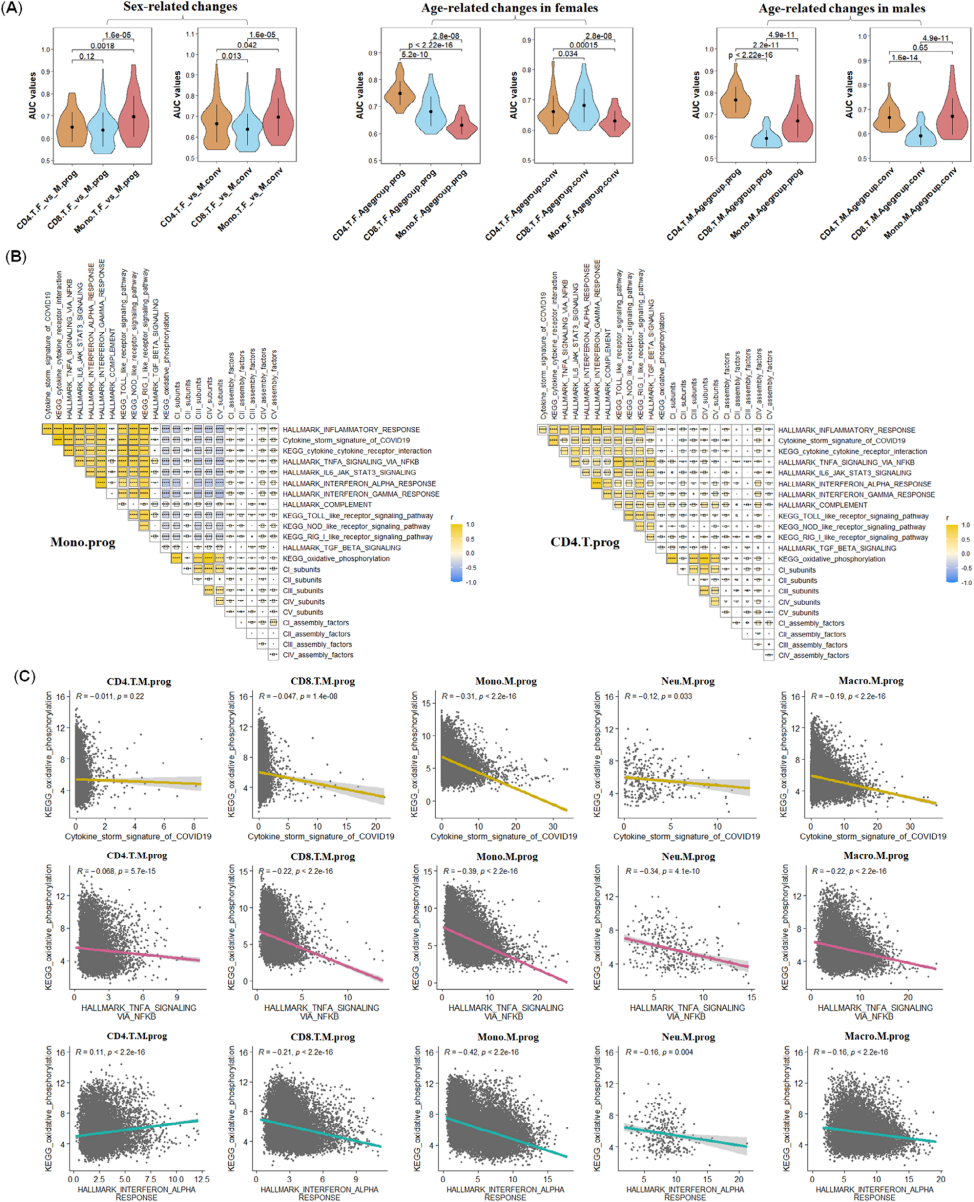
The expression changes of OXPHOS subunits in different immune cells across both genders and three comparing age groups from severe or critical COVID‐19 patients (prog). (**A**) Sex‐ or age‐related changes of mitochondrial activities derived from the accumulation of the top 20 AUC values from 149 MitoCarta pathways or gene sets in the 4 sex‐related comparing pairs, three age‐related comparing pairs of the females or the males in three types of immune cells (prog). (**B**) Correlations between mitochondrial OXPHOS subunits (including five core subunits and five assembly factors) and 11 immune pathways in mono (left panel) or CD4.T cells (right panel) from severe or critical male patients (prog) of age groups 3, 5, 6, and 7. (**C**) Correlations between three immune pathways and KEGG oxidative phosphorylation in CD4.T cells, CD8.T cells, mono, neu, and macro from severe or critical male patients of age groups 3, 5, 6, and 7.

Next, we checked whether energy metabolism was involved in the cell‐type‐dependent, age‐related, and sex‐associated variation in immunity in severe or critical COVID‐19 patients. A total of 149 mitochondrial pathways were monitored via ROC analysis. The top 20 AUC values between both genders across four age groups in three types of immune cells displayed that the mitochondrial activities in mono changed more strikingly than those in CD4.T and CD8.T (mono > CD4.T ≈ CD8.T) in severe or critical COVID‐19 patients (prog and conv) (Figure 3A, Supporting Information Figure S5, Tables [Link], [Link]), which was similar to the sex‐biased change in immune activities during disease prog (Figure 1E). The age‐related changes in mitochondrial activities were CD4.T > CD8.T > mono in female patients (prog) and CD4.T > mono > CD8.T in male patients (prog). In conv patients, the age‐related changes in mitochondrial activities were CD8.T > CD4.T > mono in females and mono ≈ CD4.T > CD8.T in males.

Notably, the KEGG oxidative phosphorylation pathway and the four OXPHOS subunits (CI, CIII, CIV, and CV) displayed stronger negative correlations with all 12 immune signaling pathways, gene sets, or signatures in Mono.prog cells than those in CD4.T.prog cells from a mixture of male and female severe or critical patients (Figure 3B, C, Supporting Information Figures S6 and S7). Other types of immune cells as CD8.T, macro, neu, DC, and NK displayed a pattern in between these two types of cells. CD4.T.conv also displayed weak negative correlations with most of the 12 immune signaling pathways, gene sets, and signatures (Supporting Information Figure S6). The negative correlations with most of the 12 immune signaling pathway, gene sets, and signatures in Mono.conv also turned weak.

Additionally, the expression profiles of KEGG oxidative phosphorylation and GOBP ATP metabolic processes along with age also displayed sex‐bias in CD8.T and mono (prog), especially in age groups 6 and 7 (Supporting Information Figure S8A). The KEGG glycolysis–gluconeogenesis pathway also displayed sex bias along with age in severe or critical patients (conv). Interestingly, a converse regulation process of OXPHOS and glycolysis in severe or critical patients during disease prog and conv from both genders was also revealed (Supporting Information Figure S8B, C). However, the positive correlation of the KEGG oxidative phosphorylation pathway with the KEGG glycolysis–gluconeogenesis pathway was usually rare in normal control and mild or moderate patients, suggesting a dynamic and disordered balance between OXPHOS and glycolysis existed in severe/critical patients (prog) (Supporting Information Figure S8D). Actually, the mutual correlations of KEGG glycolysis–gluconeogenesis, OXPHOS, and immunity were very dynamic and variable as those being shown in CD4.T, CD8.T, Mono, and Macro between both genders from severe or critical COVID‐19 patients (prog) of age group 6 (Supporting Information Figure S9).

In summary, these results suggested that cell‐type‐specific, age‐related, and sex‐dependent changes in mitochondrial activities and energy metabolism existed in immune cells from severe or critical COVID‐19 patients, which may be closely related to the cell‐type‐specific, age‐related, and sex‐biased immunity.

### 2.4 Insight of the higher immune activities of macro in male severe or critical COVID‐19 patients (60–69 years old) than the females

**FIGURE 4:**
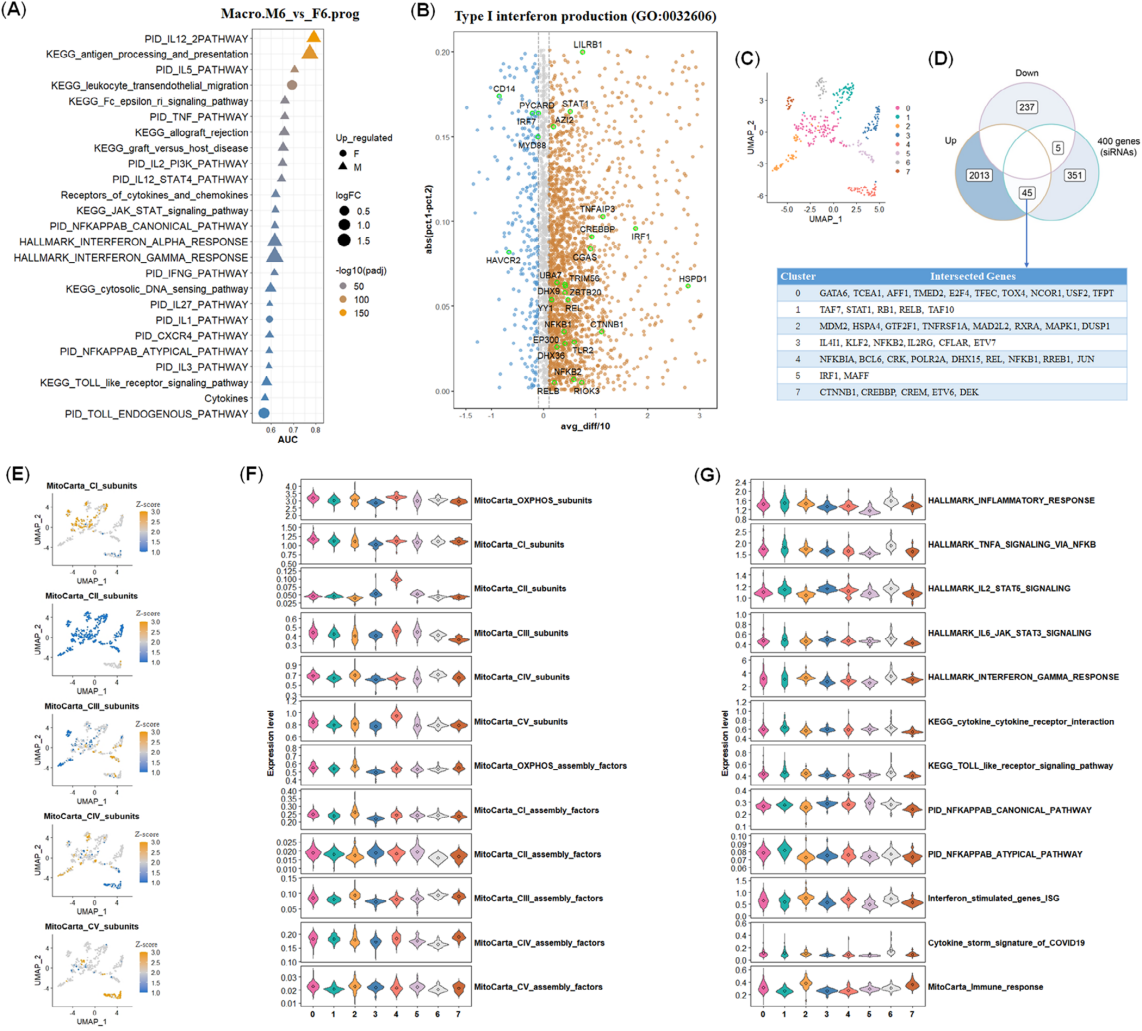
The difference of immune responses in the macro from severe or critical COVID‐19 patients (60–69 years old) between both genders and the insight of its regulation via monitoring the effects of 400 siRNAs on OXPHOS. **(A**) Top 25 changed immune pathways, gene sets, or signatures according to the ranks of AUC values between both genders from severe/critical COVID‐19 patients (60–69 years old) in prog stage (Macro.M6.prog vs. Macro.F6.prog). (**B**) The volcanic plot of differentially expressed genes enriched in type I interferon production pathway between Macro.M6.prog and Macro.F6.prog. (**C**) Uniform manifold approximation and projection (UMAP) of the 400 gene collection performing siRNA in HUVEC cells according to the OXPHOS gene expression. A total of eight cell clusters were obtained. (**D**) The intersection of prior reported 400 gene collection (siRNA in HUVEC cells) with the significantly upregulated genes and downregulated genes between Macro.M6.prog and Macro.F6.prog. The 45 genes from seven clusters, which were derived from the intersection between upregulated genes and 400 gene collection via siRNA in HUVEC cells in (**C**), were listed. (**E**) The feature plots of five subunits of OXPHOS complex. (**F**) The expression profiles of OXPHOS subunits and assembly factors in the eight clusters. (**G**) The expression profiles of 12 immune gene sets, pathways, or signatures in the eight clusters. Rhombus: mean level.

The overall immune activities in CD8.T.prog and Mono.prog from male severe or critical patients in age group 6 were usually higher than those form female patients (Figure 2B, C). We then checked whether this was also the case in macro from age group 6 just because macro usually took pivotal roles in anti‐viral immunity and age group 6 had plenty of cells across both genders (Supporting Information Figure S1B). Via pathway enrichment of differentially expressed genes (DEGs) between Macro.M6.prog and Macro.F6.prog and relative expression profile according to the ranks of AUC values in 49 immune gene sets or pathways/signatures, we revealed that immune response of macro in male severe or critical COVID‐19 patients (60–69 years old) was much higher than the females (Figure 4A, Supporting Information Table S4). For example, PID IL12‐2 PATHWAY, KEGG antigen processing and presentation, and PID IL5 PATHWAY in Macro.prog were elevated in the male patients from age group 6. Enriched DEGs showed that cytokine production was strongly induced in male Macro.M6.prog, such as type I interferon production and TNF superfamily cytokine production (Figure 4B, Supporting Information Figure S10A, B). Notably, pathways in response to steroid hormone were also strongly enriched between Macro.M6.prog and Macro.F6.prog, which may contribute to the regulation of sex‐biased immunity in macro (Supporting Information Figure S10C).

As to the changes of mitochondrial activities and the expression levels of OXPHOS subunits between Macro.M6.prog and Macro.F6.prog, we revealed that the levels of OXPHOS subunits were lowered in Macro.M6.prog than those in Macro.F6.prog, especially for CIII and CIV subunits (Supporting Information Figure S10D, Table S6), which was different from those between Macro.M6.conv and Macro.F6.conv (Supporting Information Figure S10E).

To decipher the underlying mechanism in regulating the reciprocal balance of immunity and OXPHOS, we intersected the DEGs between both genders with 400 siRNA collections in HUVEC cells,^30^ from which we could check the changes of OXPHOS and immunity in various knockdown genes (Figure 4C, D). According to the expression pattern of OXPHOS genes, the 400 siRNAs can be divided into eight clusters and the 45 genes derived from the intersection between upregulated genes and 400 gene collections via siRNA in HUVEC cells were categorized into seven clusters, suggestive of various regulation and expression patterns of OXPHOS (Figure 4D, Supporting Information Tables [Link], [Link]). The upregulated genes in the male patients, such as NFKBIA, JUN, BCL6, REL, NFKB1, CRK, DHX15, RREB1, and POLR2A, were grouped into cluster 4, which was characterized as higher MitoCarta OXPHOS subunits, CV and CII subunits, lower CI and CIV subunits, and lower immune responses when being knocked down in HUVEC cells (Figure 4E‐G). The genes from cluster 5 in increasing immunity and lowering OXPHOS were also remarkable. Though the cellular environment and gene‐regulating networks of HUVEC cells and Macro are different, but the batch reaction patterns of various genes are still referable. Notably, the distinct expression pattern of OXPHOS and immunity from the 400 siRNA also suggested that the correlation between OXPHOS and immunity may be a consequence of the regulation from multiple genes. Therefore, the elevated immunity in Macro.M6.prog was associated with the elevated expression of many of these regulatory factors.

### 2.5 The severely disordered interplay between immunity and OXPHOS in aged male severe or critical COVID‐19 patients (≥70 years old)

**FIGURE 5:**
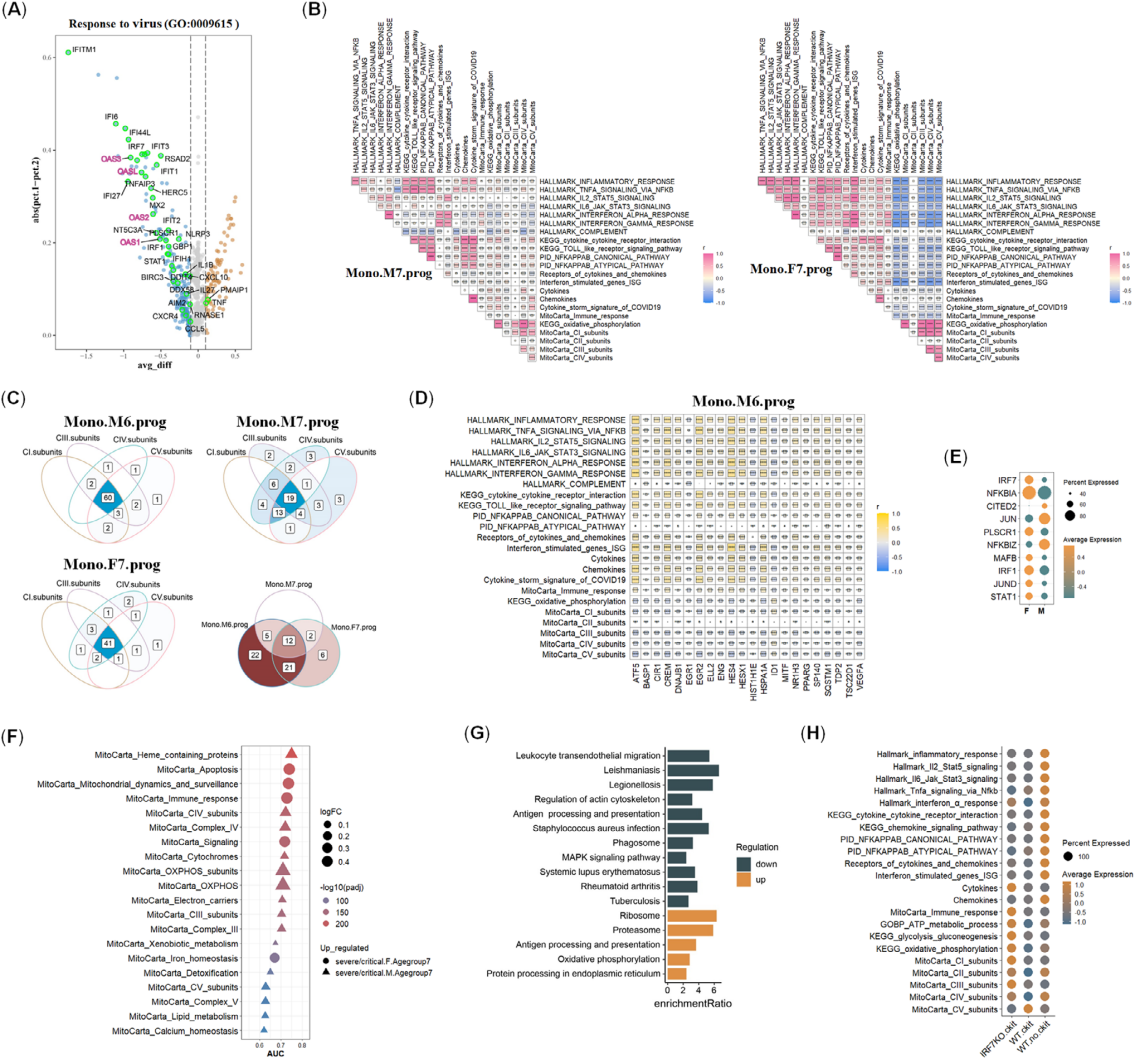
The compromised negative correlation and disordered transcriptional regulation between OXPHOS and immunity in mono from aged male severe, critical COVID‐19 patients (≥70 years old) during disease prog. (**A**) Volcanic plot of DEGs enriched in GOBP pathway Response to virus between Mono.F7.prog and Mono.M7.prog. (**B**) Correlations between mitochondrial OXPHOS subunits (including five core subunits) and 11 immune pathways in Mono.M7 (left panel) or Mono.F7 (right panel) from severe, critical male patients. (**C**) The intersection of transcription factors/cofactors (TFs) correlated with the four subunits of OXPHOS in Mono.F7, Mono.M7, Mono.M6. The mean counts of TFs > 0.2 and *p* < 0.05. The TFs were intersected further among the 60 TFs in Mono.M6, the 19 TFs in Mono.M7, and the 41 TFs in Mono.F7. **(D**) The correlation of the 23 gene sets, pathways, or signatures with the 22 unique TFs in Mono.M6. (**E**) The relative expression level of top 10 TFs between Mono.M7 and Mono.F7 according to the ranks of AUC values of 60 TFs, which was derived from the union of the 19 TFs of Mono.M7.prog and 41 TFs in Mono.M7.prog in (**D**). (**F**) The relative expression level of top 20 MitoCarta pathways according to the ranks of AUC values between Mono.M7 and Mono.F7. (**G**) KEGG enrichment pathways between IRF7ko.ckit and WT.ckit samples of AML. (**H**) The relative expression levels of 22 gene sets, pathways, or signatures among IRF7ko.ckit, WT.ckit and WT.no.ckit samples of AML.

The immune activity of male severe or critical COVID‐19 patients from age group 7 was much lower than that of female patients not only in adaptive immunity (CD4.T and CD8.T) but also in innate immunity (mono) (Figure 2A‐C). To decipher the underlying transcriptional mechanism, we performed gene differential analysis, enrichment analysis, and screening of correlated transcriptional factors/cofactors (TFs) in mono between both genders from age group 7. Pathways in cytokine production and inflammatory responses were downregulated significantly in Mono.M7.prog compared to Mono.F7.prog (Supporting Information Figure S11A, Table S10). For example, pathways in response to viruses, type I interferon, and in acute inflammatory response were compromised in Mono.M7.prog (Figure 5A, Supporting Information Figure S11B, Table S10). The important antivirus factors, such as OAS1, OAS2, OAS3, and OASL, were downregulated significantly in Mono.M7.prog. Even more, the negative correlation of the OXPHOS subunits with immune pathways or signatures was also compromised in Mono.M7.prog, comparing to Mono.F7.prog (Figure 5B). This also existed in CD4.T, CD8.T, DC, and NK cells in patients (prog) from age group 7 (Supporting Information Figure S12A). The number of significantly correlated TFs with OXPHOS subunits in Mono.M7.prog (19 TFs) was also much lesser than that in Mono.F7.prog (41 TFs) and Mono.M6.prog (60 TFs) (Figure 5C, Supporting Information Table S11), suggesting the transcriptional regulation in Mono.M7.prog was distinct from that of Mono.F7.prog and Mono.M6.prog. Actually, most of the unique 22 TFs in Mono.M6.prog and the 21 TFs shared exclusively by Mono.M6.prog and Mono.F7.prog were positively correlated with immune activities and negatively correlated with OXPHOS in Mono.M6.prog (Figure 5D, Supporting Information Figure S12B). Additionally, among these key TFs correlated with OXPHOS either in Mono.M7.prog or in Mono.F7.prog, IRF7 was unique in that it had the highest AUC values and was downregulated in Mono.M7.prog comparing to Mono.F7.prog (Figure 5E).

Notably, in line with the lower immunity, OXPHOS subunits CIII, CIV, and CV were all elevated in Mono.M7.prog in comparing to Mono.F7.prog (Figure 5F).

Next, we calculated the number of TFs correlated with the immune signature of interferon‐stimulated genes in Mono.M6, Mono.M7, and Mono.F7 (Supporting Information Figure S12C, Table S11). A total of 13 TFs were unique in Mono.M6.prog and 19 TFs were shared exclusively by Mono.M6.prog and Mono.F7.prog, among which the vast majority were also positively correlated with immune activities and negatively correlated with OXPHOS in Mono.M6.prog (Supporting Information Figure S12D).

To further elucidate the disordered regulation of immune responses and mitochondrial OXPHOS in Mono.M7.prog, we checked the role of IRF7 with IRF7‐knockout and MLL‐AF9‐induced acute myeloid leukemia (AML) mouse models.^31^ KEGG enrichment analysis of the DEGs indicated that immune responses were downregulated and OXPHOS was upregulated in IRF7ko.ckit sample comparing to WT.ckit sample (Figure 5G). The upregulation of OXPHOS was much prominent in IRF7ko.ckit sample and ckit (KIT) depletion considerably improved the overall immune activities in AML (Figure 5H). Therefore, the downregulation of IRF7 in Mono.M7 may be one contributing factor resulting in the uncoupled immune responses and mitochondrial OXPHOS in Mono, which may account for the bias of more morbidity in aged male severe or critical COVID‐19 patients. Additionally, ckit may participate in regulating the coupling of OXPHOS and immunity in myeloid cells.

In summary, the lower immunity in Mono.M7.prog than that in Mono.F7.prog or Mono.M6.prog was highly related to the dysregulated TFs, which were usually involved in improving immune activities and lowering OXPHOS. Additionally, in myeloid cells, genes as IRF7 and KIT, were also needed in the coupling of downregulation of OXPHOS with the increase in immunity.

### 2.6 The positive correlation of testosterone with OXPHOS in immune cells from a castration and regeneration mice model

**FIGURE 6:**
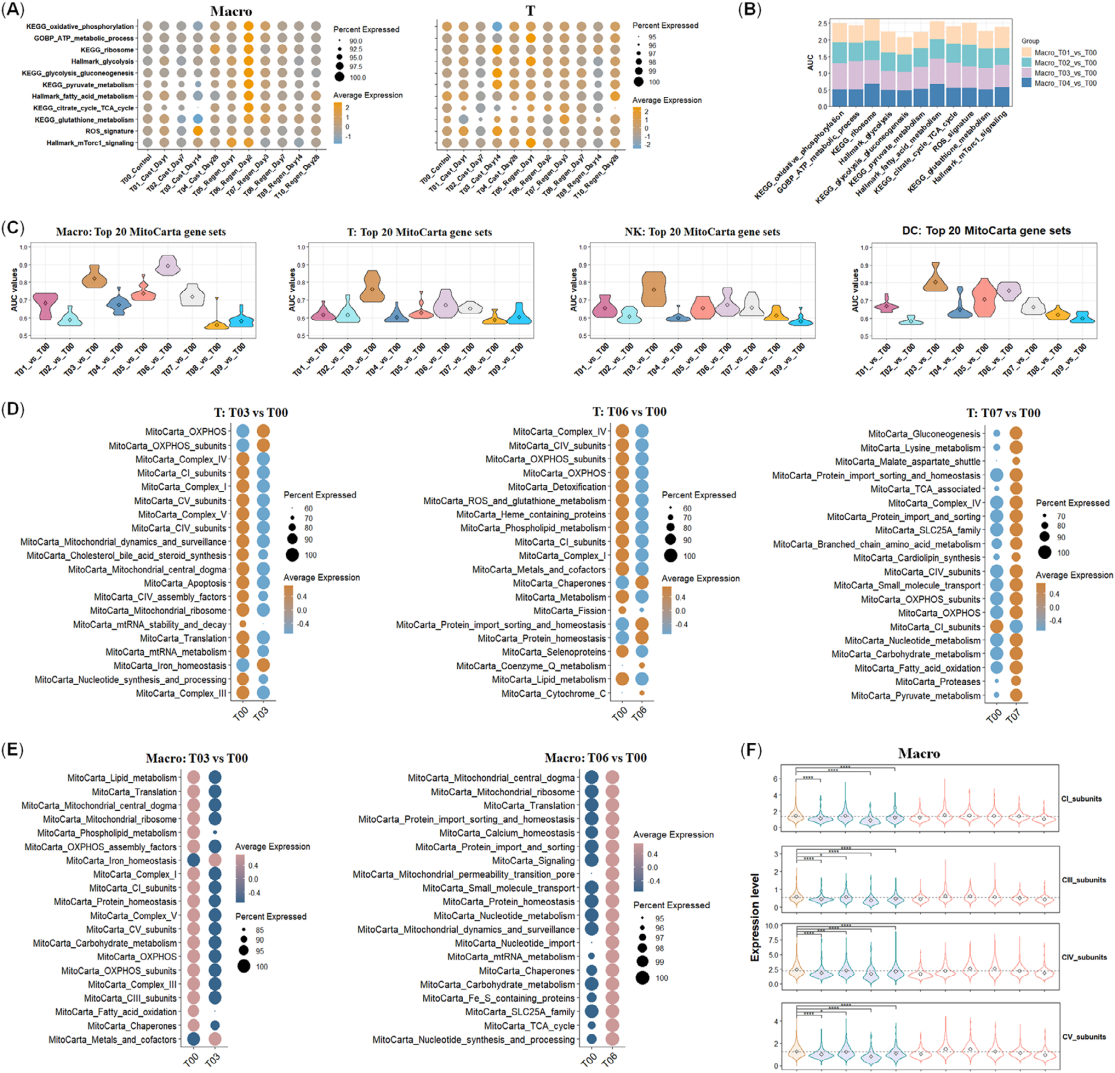
The dynamic fluctuation of mitochondrial activities and OXPHOS expression levels in response to androgen deprivation and regeneration in immune cells. (**A**) Expression dynamics of 11 metabolic pathways in macro (left panel) and T cells (right panel) from castration prostates and regeneration prostates. **(B**) Stacked AUC values of 11 metabolic pathways across four comparison groups (T01 vs. T00, T02 vs. T00, T03 vs. T00, T04 vs. T00) in prostate Macro by ROC analysis. (**C**) Top 20 AUC values of 149 MitoCarta pathways, gene sets in four types of immune cells in nine pairwise comparisons of castration prostates (T01, T02, T03, and T04) (days 1, 7, 14, and 28) and regeneration prostates (T05, T06, T07, T08, and T09) (days 1, 2, 3, 7, and 14) with normal control (hormonally intact samples, T00). **(D‐E**) The relative expression levels of the top 20 changed MitoCarta pathways, gene sets in three compared groups of T cell, two compared groups in macro, and two compared groups in NK cells according to the ranking of AUC values. (**F**) Expression dynamics of the four core subunits of mitochondrial OXPHOS in Macro (left panel) and T cells (right panel) of castration prostates or regeneration prostates. One‐sided Wilcoxon rank‐sum test was performed. Only the significance between the castrated samples and the control is labeled. Mean levels were indicated with dashed lines.

A lower level of serum testosterone is common in aging men.^32^ Sex hormone milieus should play key roles in determining the sex dimorphism of immunity, though it still needs more evidences.^5^ Androgen is also well known for its prominent roles in regulating metabolism and the sex dimorphism of metabolic diseases.^33^, ^34^ Castration and regeneration animal models with large cell samples were ideal for such an investigation. To monitor the role of androgen in metabolism and immunity, we then compared the expression profiles of 11 representative metabolism‐related pathways or signatures including the OXPHOS pathway of macro and T cells in a castration and regeneration mice model published previously.^35^ The expression profiles of these pathways or signatures displayed distinct patterns in macro and T cells (Figure 6A). For example, most metabolism‐related pathways of Macro on day 2 postregeneration reached peaks that were not found in T cells, and only three pathways reached the maximum levels on day 1 postregeneration. Among the 11 pathways or signatures, the oxidative phosphorylation pathway decreased gradually during the first 14 days postcastration in both macro and mixed T cells and may be a candidate marker of androgen deprivation. Next, ROC analysis was conducted and verified that most metabolism‐related pathways or signatures had good classification accuracy in representing the changes in macro within 14 days postcastration, among which, KEGG oxidative phosphorylation, GOBP ATP metabolic process, KEGG ribosome, and ROS signatures performed the best (Figure 6B). According to the ranks of the top 20 AUC values in the nine comparing pairs, we revealed that T03 (on day 14 postcastration) had the most striking fluctuation in MitoCarta gene sets or pathways in the four types of immune cells (Figure 6C, Supporting Information Table S12). Similar to the immune responses in macro on day 2 postregeneration (T06), mitochondrial activities changed most prominently than those in other three types of immune cells, further suggesting macro was more sensitive to androgen fluctuation.

Even more, androgen deprivation led to inhibition of mitochondrial activities in T cells and macro (Figure 6D, E, Supporting Information Table S13). The levels of CI, CIV, and CV subunits of mitochondrial OXPHOS were usually downregulated. More interestingly, the inhibition was restored postregeneration, but the response of T cells was slower than macro, which was recovered on day 2 (T06) postregeneration and on day 3 (T07) in T cells. In neu, the level of OXPHOS subunits was usually higher in young adult males mice than female mice, and castration reversed this trend (Supporting Information Figure S13A, Table S14). The levels of the four core subunits of mitochondrial OXPHOS complexes in macro were decreased in most castration samples (Figure 6F), which was in line with prior reports that androgens regulate the level of OXPHOS and ATP production in mitochondria.^36^, ^37^, ^38^, ^39^ These data demonstrated that though the dynamic response of OXPHOS to testosterone varied in different types of immune cells, both displayed a positive correlation pattern.

### 2.7 The strong negative correlation of OXPHOS with immunity in mouse macro and neu

**FIGURE 7:**
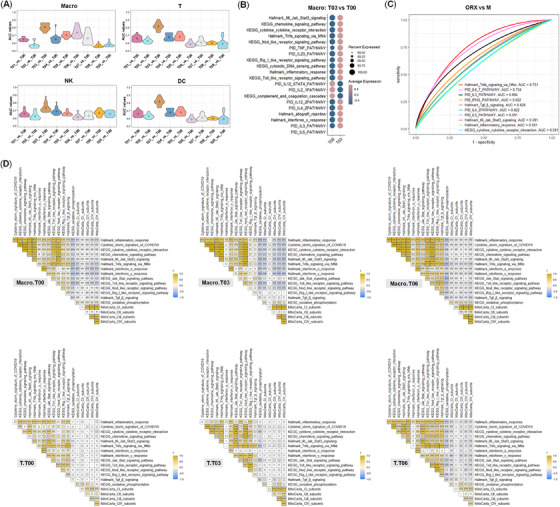
The triple interplay of testosterone, OXPHOS and immunity. (**A**) Top 20 AUC values of 40 immune pathways, gene sets, signatures in four types of immune cells in nine pairwise comparisons of castration prostates at four timepoints (days 1, 7, 14, and 28) and regeneration prostates at five timepoints (days 1, 2, 3, 7, and 14) with normal control prostates (hormonally intact samples, T00). (**B**) The relative expression level of T03 versus T00 in macro according to the ranks of the top 20 AUC values of 40 immune pathways, gene sets. (**C**) The smoothed ROC curves of top 10 immune pathways in mature neu according to the ranks of unsmoothed AUC values between orchiectomy sample (ORX) and the normal adult male mice. (**D**) The correlations between OXPHOS and 12 immune pathways in macro and T cells in T00, T03, (castration day 14) and T06 (regeneration day 2) samples.

According to the ranks of the top 20 AUC values in the nine comparing pairs, we revealed that T03 (on day 14 postcastration) had the most striking fluctuation in immune activities in the four types of immune cells (Figure 7A, Supporting Information Table S12). More importantly, the immune fluctuation in macro on day 2 postregeneration (androgen add‐back, T06) was most considerably among the four types of immune cells, suggesting fluctuation of androgen level has more impact on macro than other three types of immune cells, which was also the same to mitochondrial activities in macro (Figure 6C). According to the ranks of AUC values, the top 20 immune pathways in macro (T03_vs_T00) and the other four comparing groups in both macro and T were also displayed (Figure 7B, Supporting Information Figure S14, Table S15), indicative of the immune suppressive effects of androgens.^40^

According to the correlation patterns among immune responses, OXPHOS, and androgen in immune cells, strong negative correlation was revealed in macro, which was also the same in mature neu (Figure 7C, D, Supporting Information Figure S13B, Table S14). CI, CIII, CIV, and CV subunits of OXPHOS, KEGG oxidative phosphorylation, were negatively correlated with most of the 12 immune pathways in macro from intact control (T00), castration (T03), and regeneration sample (T06). The correlation patterns in T, NK, and DC were relatively weak in mice (Figure 7D, Supporting Information Figure S15A). However, in DC, a transient negative correlation of immune responses and OXPHOS was found on day 1 postcastration (T01) and then this correlation pattern turned very weak in other treatment groups. Notably, the negative correlation between immune pathways, gene sets, and signatures and KEGG oxidative phosphorylation existed in the control sample, all castration samples, and all regeneration samples of macro (Supporting Information Figure S15B). Therefore, cell‐type‐specific regulatory coupling of the expression of OXPHOS with immunity also existed in immune cells of mice, and the negative correlation of both in some cell types from myeloid cells was usually more prominent.

## 3 DISCUSSION

The metabolic program in immune cells is well known to be changed along with pro‐ or anti‐inflammatory responses, a process also known as immunometabolism.^41^ Krebs cycle intermediates, glycolysis, fatty‐acid synthesis, and beta oxidation are highly related to the M1 or M2 polarization in Macro.^42^, ^43^ The mitochondrial metabolic program and immunity also varied in different types of immune cells and subsets.^44^ The level of OXPHOS may reflect the reciprocal levels of glycolysis and tricarboxylic acid cycle within immune cells, which determine the proinflammatory or anti‐inflammatory states (including resting states). In this context, one important role of androgen is likely to inhibit the glycolysis and increase the OXPHOS level to modulate the immunity. Therefore, the negative correlation of OXPHOS with immunity may be important for the normal immune responses. As a negative example, the compromised negative correlation between OXPHOS and immunity in the male severe or critical COVID‐19 patients (prog) from age group 7 may be an important contributing factor leading to highest vulnerability to morbidity and mortality.

Both of the age‐related increase of immune activity and the cell‐type‐ or age‐related decline of OXPHOS in male severe or critical COVID‐19 patients were similar to those in castrated animals. For example, compared to age group 3, age groups 5 and 6 of CD8.T and Mono showed higher immune activities in the male severe or critical COVID‐19 patients (prog) (Figure 2B, C). This is in line with the hypothesis that the immunity in adult and aged males increased steadily along with androgen decline gradually with age, which is evidenced in prior reports that the immune activities of innate immune cells increased with aging, and the activation magnitude was more pronounced in men.^28^, ^45^ More strikingly, these studies indicated a significant sex‐biased enrichment in open loci of chromosomes towards mono and DC in aged males and toward T cells and B cells in aged females. Therefore, older females tended to have higher genomic activities in adaptive immune cells, and older men tended to have higher genomic activities in mono.^28^ It is thus in line with this sex dimorphism in immunity that females are more vulnerable to autoimmune disorders and men, who have higher proinflammatory cytokine production, are more susceptible to infectious diseases.^4^ Therefore, in male severe or critical COVID‐19 patients, age groups 5 and 6 had a higher potential for suffering from cytokine storms, and age group 7 had a higher probability of suffering from hypoinflammation than the females.

Among the underlying mechanisms accounting for the sex dimorphism in immune activities, the distinct roles of androgens and estrogens at physiological concentrations are important.^46^ For example, evidence supports the immunosuppressive activities of androgens and immunostimulatory activities of estrogens.^4^, ^18^, ^47^, ^48^, ^49^ This was also strongly evidenced by the survival rates of ovariectomized female mice or castrated male mice after lethal infection.^50^, ^51^ Notably, androgen deprivation may increase the basal immune activities of some immune cell types and thus potentially exert synergistic effects with infection.^52^, ^53^, ^54^ This synergistic effect in severe or critical COVID‐19 patients potentially aggravates cytokine storms in men and leads to the life‐threatening phase of acute respiratory distress syndrome, as more and more evidence suggests that cytokine storms are mainly a consequence of the elevated activities of innate immune cells.^16^, ^55^ During this process, adaptive immune activities decline and innate immune responses are elevated, within which mono, macro, and neu are more relevant to severe outcomes.^56^, ^57^ COVID‐19 patients with hyperinflammation are more vulnerable to poor outcomes than those with hypoinflammation.^58^, ^59^ In this study, the males from age groups 5 and 6 were more likely suffered from hyperinflammation and those from age group 7 more likely developed hypoinflammation than the females. Since the aged male severe or critical patients are more vulnerable to late‐onset hypogonadism due to advanced age,^32^, ^60^ testosterone replacement therapy is, therefore, more dangerous for the groups of patients than other age groups due to the strong immune suppressive response of macro to testosterone replacement (Figure 7B, Supporting Information Figure S14). Though the elevated immunity, especially in age groups 5 and 6, seemed to be related to the decline of testosterone in the male patients, the deficiency in immunity in severe or critical male patients from age group 7 was likely associated with accelerated aging in immunity. Unfortunately, due to the lack of information about androgen levels in serum from this cohort of COVID‐19 patients, the explanation of age‐related immunity and androgen levels in the males was still limited. More experiments are needed for further validation in future investigation.

As to the mechanism behind the biased effect of androgens on innate and adaptive immune cells, there is still no clear answer. In this report, our finding of the suppressive role of androgens on oxidative phosphorylation in mitochondria was striking and is similar to the well‐known suppressive effect of inflammation on oxidative phosphorylation in immune cells.^25^, ^43^ According to the putative interplay between androgen signals and inflammation stimulus signals, we propose that the electron transport chain (ETC) in mitochondria may be a hub of convergence for both androgen deprivation and inflammation stimulus signals that drive immune cell polarization and immune activation. For example, upon M1 macrophage stimuli by LPS or IFN‐gamma, decreased respiration and disruption of the Krebs cycle result, in the accumulation of succinate and citrate, which stabilize HIF1A to trigger HIF1A‐IL1B axis signaling.^61^, ^62^ An increase in the ΔΨm, accumulation of succinate, and the resultant reversion in electron transport at complex I of ETC in M1 Macro also result in the increased production of oxidants, which is a likely reason for the activation of the HIF1A‐IL1B axis.^63^, ^64^ ARs bear a mitochondrion localization sequence, and the expression level of genes from complexes II, III, and IV of ETC and the activity of complex III were increased by knocking‐down ARs.^36^ ARs directly regulate the catalytic subunits (SDHA and SDHB) of succinate dehydrogenase via androgen response elements, and inhibition of ARs leads to the accumulation of succinate.^65^ Furthermore, androgen deficiency was revealed to increase ROS production.^66^, ^67^, ^68^ All three key features of the impact of AR inhibition or androgen deprivation on ETC are similar to the effects of stimulus by LPS and interferon on macro, which may account for the similar immune activation coordinated via the HIF1A‐IL1B axis. Additionally, the direct roles of androgen on NRF1‐mediated transcription, AR‐PGC‐1α/PGC‐1β‐mediated transcription on oxidative phosphorylation‐related members, and the global metabolic regulation of AR via CAMKK2‐AMPK signaling or mTOR signaling have also been proposed to have roles in oxidative phosphorylation.^69^, ^70^

In summary, sex bias in immunity and energy metabolism were important features in shaping the adaptive and innate immunity in severe or critical COVID‐19 patients, which usually displayed cell‐type‐dependent and age‐related characteristics between both genders. In advanced age, the sex bias was extremely prominent. Therefore, sex and age factors should be given particular attention in samples involved in immunity and metabolism.

## 4 MATERIAL AND METHODS

### 4.1 Single‐cell RNA‐seq data processing, batch effect correction, and cell subset annotation

The scRNA‐seq data for intact prostate, castration prostate, and regeneration prostate were derived from the NCBI GEO database: GSE146811. The deposited raw count data were processed, and all cell clusters were annotated according to an original report.^35^ Batch effect correction was performed by the SCTransform function built in Seurat V4.^71^ Because of lower cell viability, FACS‐isolated cells were discarded. Cell barcode information is listed in Supporting Information Table S16.

The scRNA‐seq data of mature neu from bone marrow were derived from the NCBI database accession number GEO: GSE198175.^72^ The three treated samples were integrated (nfeatures = 2000) with Seurat V4. Mature neu from both genders of normal mature mice and castrated mice (ORX) were identified according to the markers Csf3r, Il1b, Ccl6, C5ar, and Retnlg. The Batch effect of the raw count data of mature neu was removed with the SCTransform function in Seurat V4. Cell barcode information is listed in Supporting Information Table S17.

The scRNA‐seq data of COVID‐19 patients were derived from the NCBI GEO database: GSE158055. The raw count data and meta.data annotating the cell clusters were used according to the original report.^27^ The patients were divided into groups according to age: 3 (30−39 years old), 4 (40−49 years old), 5 (50−59 years old), 6 (60−69 years old), and 7 (≥70 years old). Batch effect correction was performed separately for each major cell type by R package Harmony. For Macro, the batch effect correction was performed by the SCTransform function in Seurat V4.

### 4.2 Bulk RNA‐seq data processing

A total of 400 siRNAs data in HUVEC cells were from GEO database with accession code GSE27869.^30^ Dimensionality reduction was performed on the normalized data with UMAP with the genes from MitoCarta_OXPHOS to obtain eight clusters among the 400 siRNA. The scores of gene sets were calculated as counts of each gene set or counts of all genes × 100.

Bulk RNA‐seq data of IRF7KO and WT were obtained from GEO database with accession code GSE178560.^31^ The raw data were normalized. The scores of gene sets were calculated as counts of each gene set/counts of all genes × 100.

### 4.3 Pathways, signatures, TFs, and TF cofactors

Gene sets and pathways were copied from Hallmark gene sets of MSigDB collections, the KEGG pathway database, Reactome pathway database, and the WikiPathways pathway database, and some ontology terms were derived from the Gene Ontology resource. OXPHOS gene signatures were from MitoCarta3.0.^73^ Cytokine storm signature of COVID‐19 and other signatures were from prior reports and are listed in Supporting Information Table S18.^27^, ^74^, ^75^ Pathways, gene sets, and signatures from scRNA_seq were evaluated with the PercentageFeatureSet function built into Seurat V4.3.

TFs and TF cofactors were obtained from the database of AnimalTFDB 3.0.^76^ TFs and TF cofactors were further filtered with mean counts more than 0.2.

### 4.4 Statistical analysis, correlation analysis, and ROC analysis

All statistical analyses were performed with unpaired Wilcoxon rank‐sum test (two‐sided or less) with Bonferroni correction via the rstatix package or ggpubr package.

Pearson correlation coefficient was calculated with the linkET package (*p* < 0.05).

AUC values were calculated with the wilcoxauc function with unsmoothed method in R package presto. AUC plotting was performed with a binormal smoothing method with the ggroc function built into the R package pROC, which values were a little bit different to those of unsmoothed values (Figure 7C).^77^ Ranks of AUC values were in descending order.

### 4.5 Differential expression and pathway enrichment analysis

DEGs between Mono.M7.prog and Mono.F7.prog were identified via FindMarkers (test.use = wilcox, min.pct = 0.15, logfc. threshold = 0.25, avg_diff > 0.1 or < −0.1) in Seurat 4.3. DEGs between Macro.M7.prog and Macro.F7.prog were performed with (test.use = wilcox,, min.pct = 0.25, logfc. threshold = 0.25, avg_diff > 1 or < ‐1). Upregulated or down‐regulated genes were then enriched in nonredundant pathways via WebGestalt and filtered with FDR < 0.05.^78^ The enriched pathway terms were further plotted with R package simplifyEnrichment to generate the word cloud heatmap of enriched pathways.

## AUTHOR CONTRIBUTIONS

Conceptualization, YL, RL, FS, SJ; methodology, YL, LL, GW; software, GX, GW; validation, YL, SJ, LY, JC, SL; formal analysis, YL; resources, GX, LL, JZ, YH, JC, FS, SL, RL; visualization, YL, LL, SJ, GX; writing—original draft, YL, SJ; writing—review & editing, RL, FS, SJ, LY; supervision, SJ, FS, RL; project administration, FS, RL; funding acquisition, RL, FS, YL. All authors have read and approved the final manuscript.

## CONFLICT OF INTEREST STATEMENT

The authors have no conflict of interest to declare.

## ETHICS APPROVAL STATEMENT

Not applicable.

## Supporting information

Supporting Information

Supporting Information

Supporting Information

Supporting Information

Supporting Information

Supporting Information

Supporting Information

Supporting Information

Supporting Information

Supporting Information

Supporting Information

Supporting Information

Supporting Information

Supporting Information

Supporting Information

Supporting Information

Supporting Information

## Data Availability

All data were earlier reported and accessible freely from GEO database (GSE158055, GSE146811, GSE198175, GSE27869, and GSE178560).
