# Supplementary material for: The unique interplay of mitochondrial oxidative phosphorylation (OXPHOS) and immunity and its potential implication for the sex‐ and age‐related morbidity of severe COVID‐19 patients
Source: MedComm (2020). 2023 Sep 23;4(5):e371. doi: 10.1002/mco2.371 (PMC10518039; doi:10.1002/mco2.371)
Supplement: Supplementary file 2 — Supporting Information [file MCO2-4-e371-s005.pdf]

The unique interplay of mitochondrial oxidative phosphorylation (OXPHOS) and immunity and it's potential implication for the sex- and age-related morbidity of severe COVID-19 patients

Yinchuan Li[1#], Lei Li[2#], Guanghao Wu[3#], Gangcai Xie[1], Lirong Yi[1], Jie Zhu[4,5], ShiYu Liang[4], Ya-ru Huang[4], Juan Chen[2], Shaoyang Ji[4]*, Fei Sun[1]*, Rui-tian Liu[4]*

Affiliations:

[1]Institute of Reproductive Medicine, Medical School of Nantong University, Nantong, Jiangsu 226001, China.

[2]National Clinical Research Center for Obstetric & Gynecologic Diseases, Department of Obstetrics and Gynecology, Peking Union Medical College Hospital, Chinese Academy of Medical Sciences & Peking Union Medical College, Beijing 100730, China.

[3]School of Materials Science and Engineering, Beijing Institute of Technology, Beijing 100081, P.R. China.

[4]National Key Laboratory of Biochemical Engineering, Institute of Process Engineering, Chinese Academy of Sciences, Beijing 100190, China.

[5]University of Chinese Academy of Sciences, Beijing 100049, China.

# These authors contributed equally: Yinchuan Li, Lei Li, and Guanghao Wu.

* Corresponding author. Email: jishaoyang@ipe.ac.cn; sunfei@ntu.edu.cn; rtliu@ipe.ac.cn

Supplementary Information

Severity	Agegroup1	Agegroup2	Agegroup3	Agegroup4	Agegroup5	Agegroup6	Agegroup7
mild/moderate	**8**	**3**	**18**	**18**	**11**	**3**	**5**
severe/critical	**0**	**0**	**6**	**4**	**15**	**17**	**13**

(X^2 = 39.204, df = 6, p = 6.528e-07)

Supplementary Table 2. Pearson's Chi-squared test in mild/moderate and severe/critical COVID-19 patients among 7 age groups.

Gender	Agegroup1	Agegroup2	Agegroup3	Agegroup4	Agegroup5	Agegroup6	Agegroup7	counts	M/F
F	**0**	**0**	**2**	**0**	**8**	**6**	**4**	**20**	**1.75**
M	**0**	**0**	**4**	**4**	**7**	**11**	**9**	**35**	

Supplementary Table 3. Gender ratio among severe COVID-19 patients.

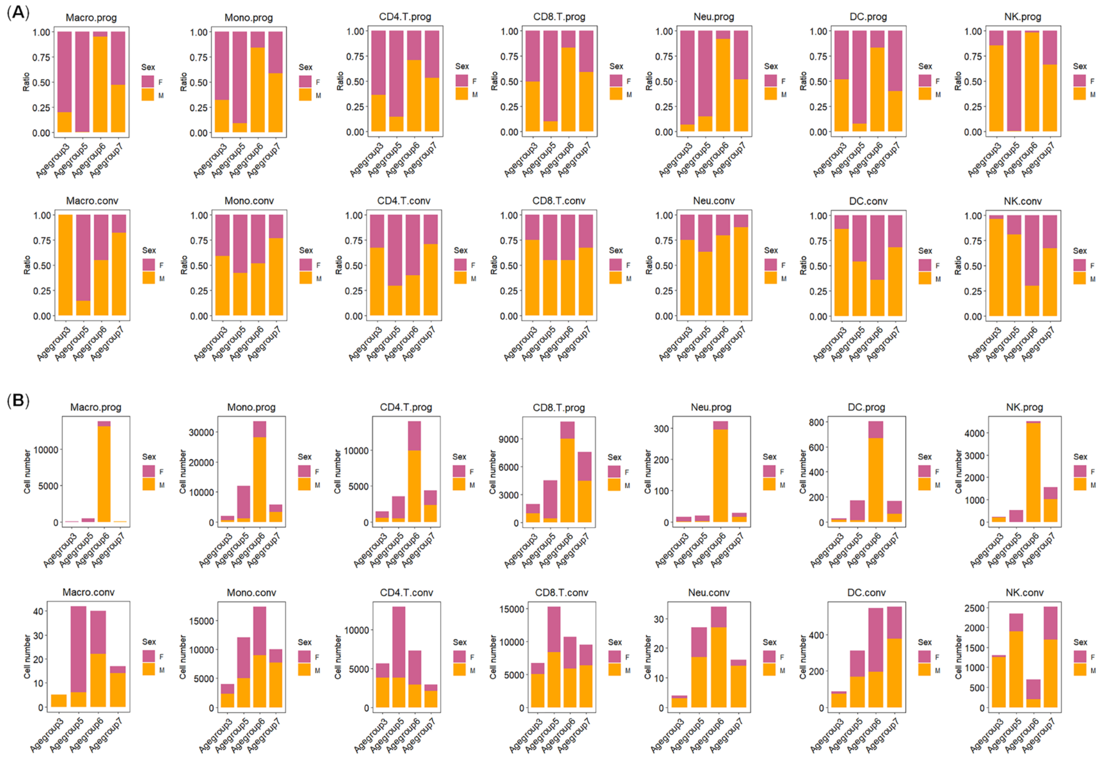

Supplementary Figure 1.

Cell proportions (upper panels) and cell numbers (lower panels) of seven types of immune cells from both genders of severe/critical COVID-19 patients (prog or conv). (**A**) Cell proportions from four age groups of patients during disease prog (upper panels) and conv (lower panels) in 7 types of immune cells between both genders. (**B**) The cell numbers of 7 types of immune cells in 4 age

groups from severe/critical patients (prog or conv) between both genders as in (**A**).

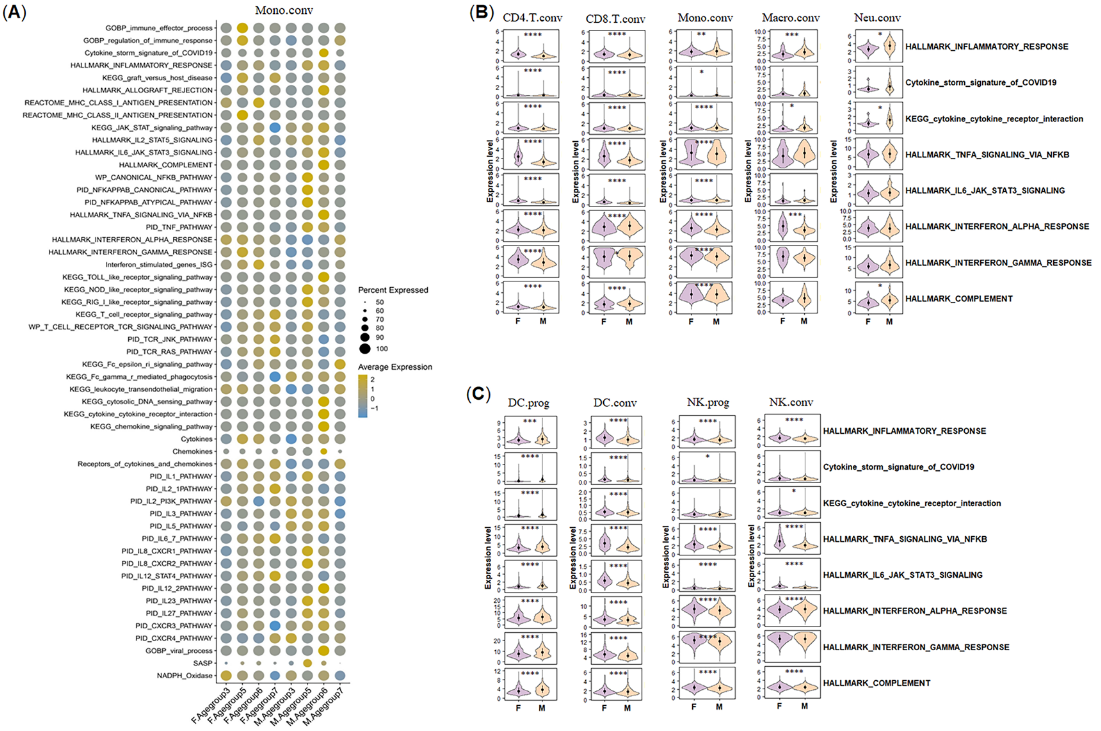

Supplementary Figure 2.

The expression profiles of immune pathways in immune cells from severe/critical COVOD-19 patients (conv). (**A**) The expression profiles of 49 immune pathways/gene sets/signatures in Mono.conv across 4 age groups of severe/critical patients between both genders. (**B**) Relative expression profiles of eight immune pathways between both genders in 5 types of immune cells from severe/critical patients (conv). (**C**) Relative expression profiles of eight immune pathways between both genders in 2 types of immune cells from severe/critical patients (prog and conv). Wilcoxon rank-sum test was performed with Bonferroni correction. *p < 0.05, **p < 0.01, ***p < 0.005, ****p < 0.001.

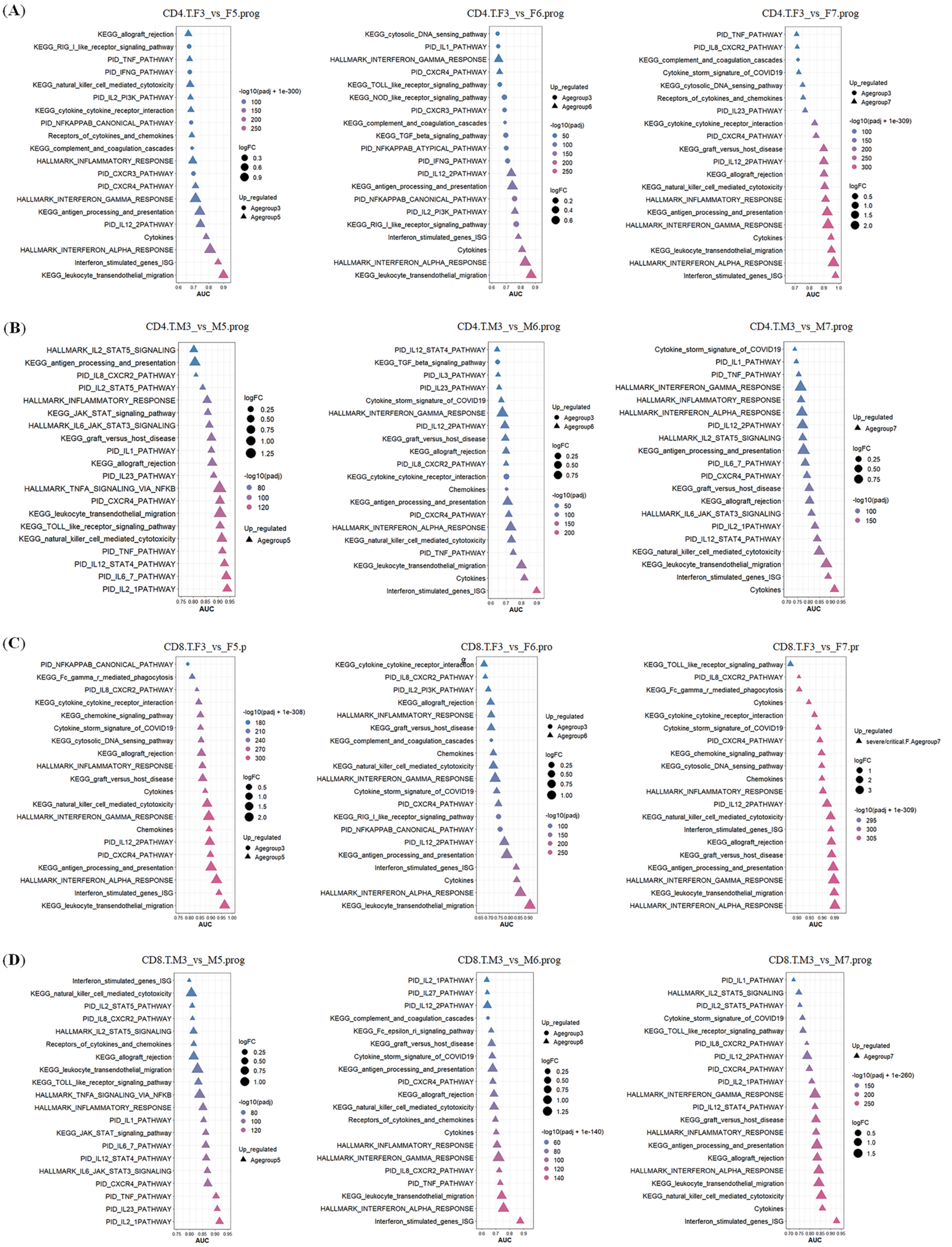

Supplementary Figure 3.

Age-related changes in immune activities of CD4.T cells and CD8.T cells (prog) in severe/critical COVID-19 patients. The top 20 changed immune pathways/gene sets/signatures according to the ranks of AUC values in 3 comparing age groups from male or female severe/critical COVID-19 patients (prog) in CD4.T cells (F) (**A**), CD4.T cells (M) (**B**), CD8.T cells (F) (**C**), and CD8.T cells (M) (**D**).

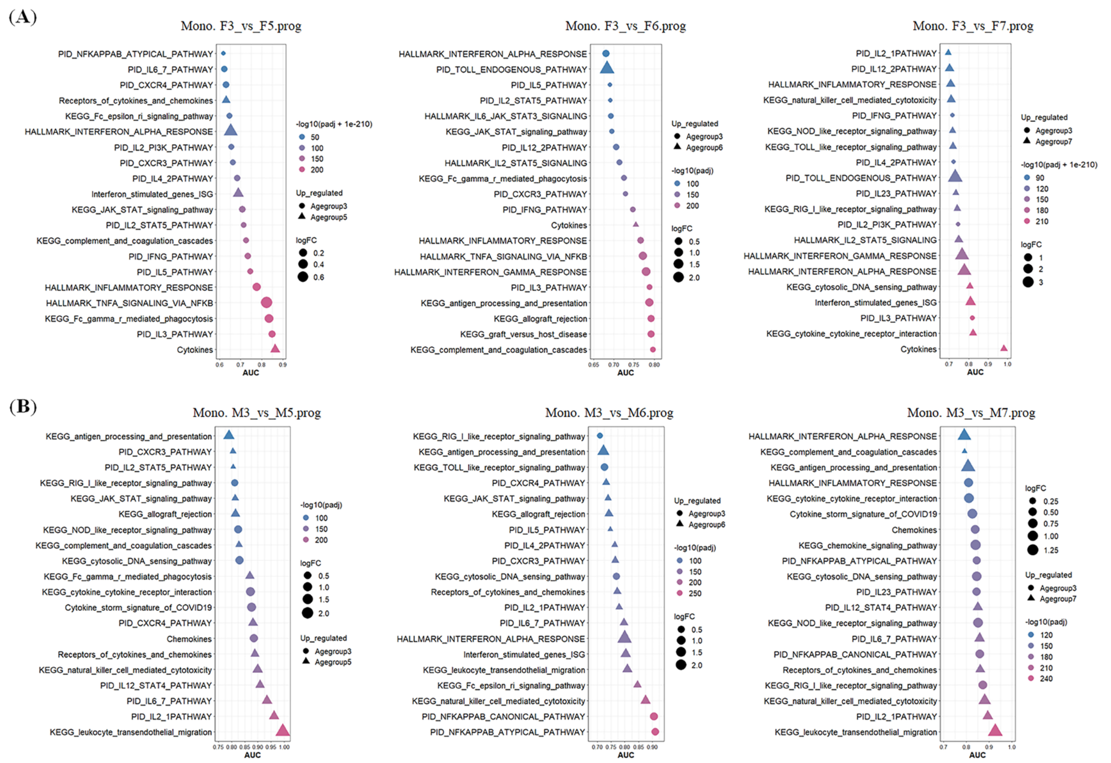

Supplementary Figure 4.

Age-related changes in immune activities of Mono (prog) from severe/critical COVID-19 patients. The top 20 changed immune pathways/gene sets/signatures according to the ranks of AUC values in 3 comparing age groups in Mono from female patients (prog) (**A**) or male patients (prog) (**B**).

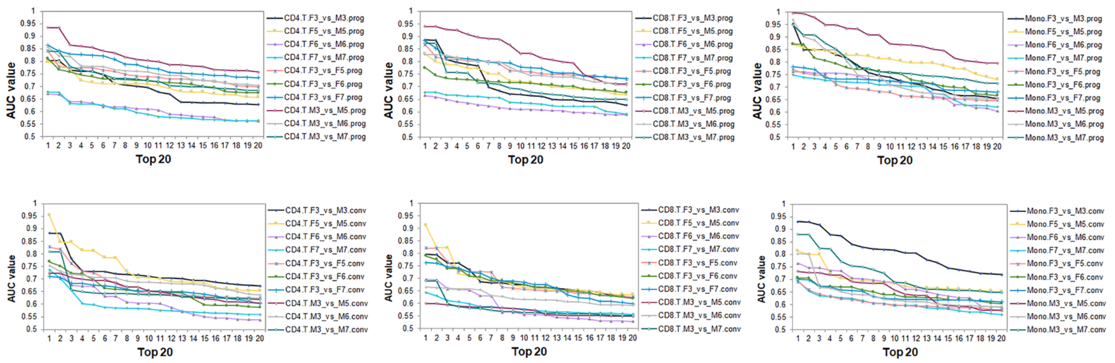

Supplementary Figure 5.

Sex- or age-related changes of mitochondrial activities derived from the accumulation of the top 20 AUC values from 149 MitoCarta pathways/gene sets in the 4 sex-related comparing pairs, 3 age-related comparing pairs of females or males in 3 types of immune cells in sever/critical COVID-19 patients during disease prog (upper panels) or conv (lower panels).

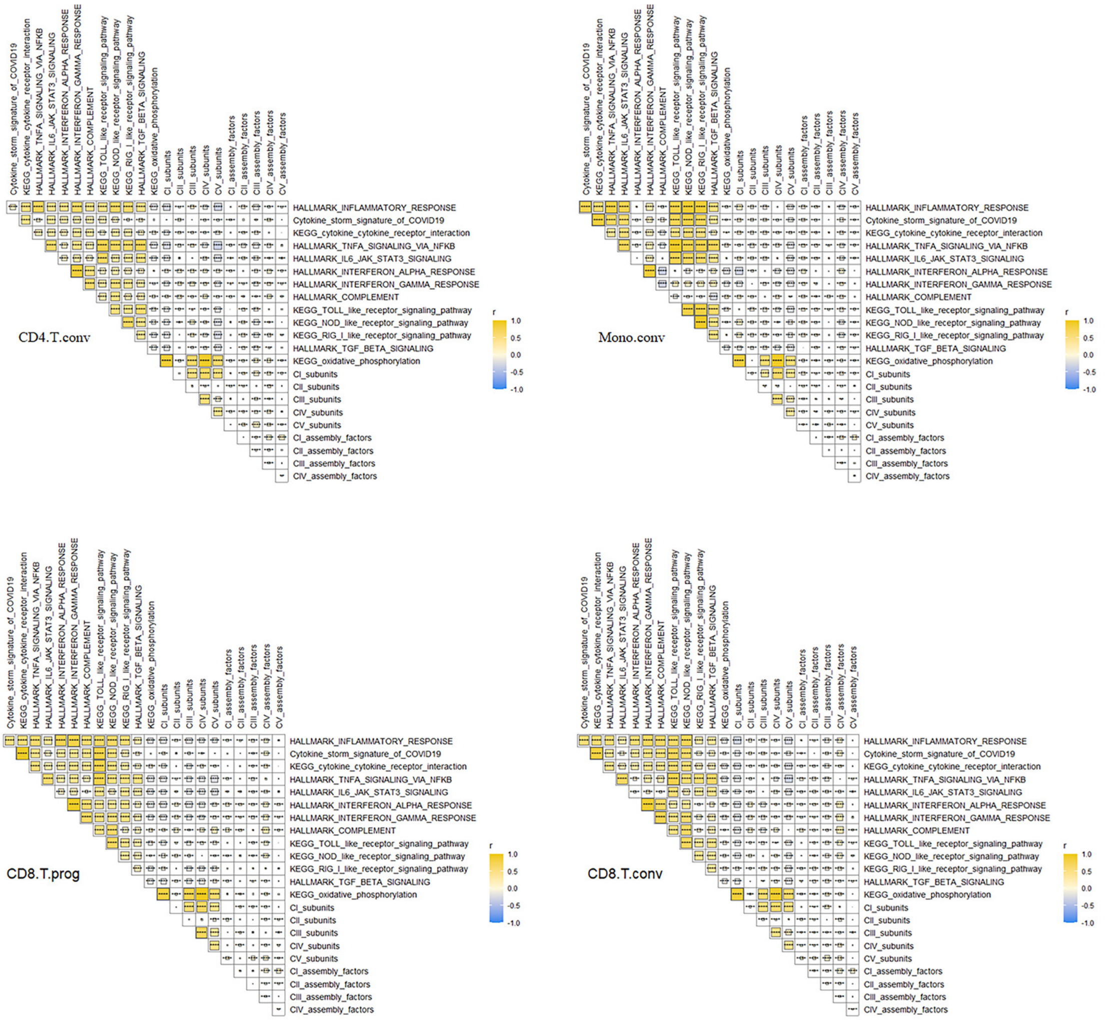

Supplementary Figure 6.

Correlations between mitochondrial OXPHOS subunits (including five core subunits and five assembly factors) and 11 immune pathways in CD4.T.M.conv, Mono.M.conv, CD8.T.M.prog and CD8.T.M.conv from male severe/critical patients of age groups 3, 5, 6, and 7.

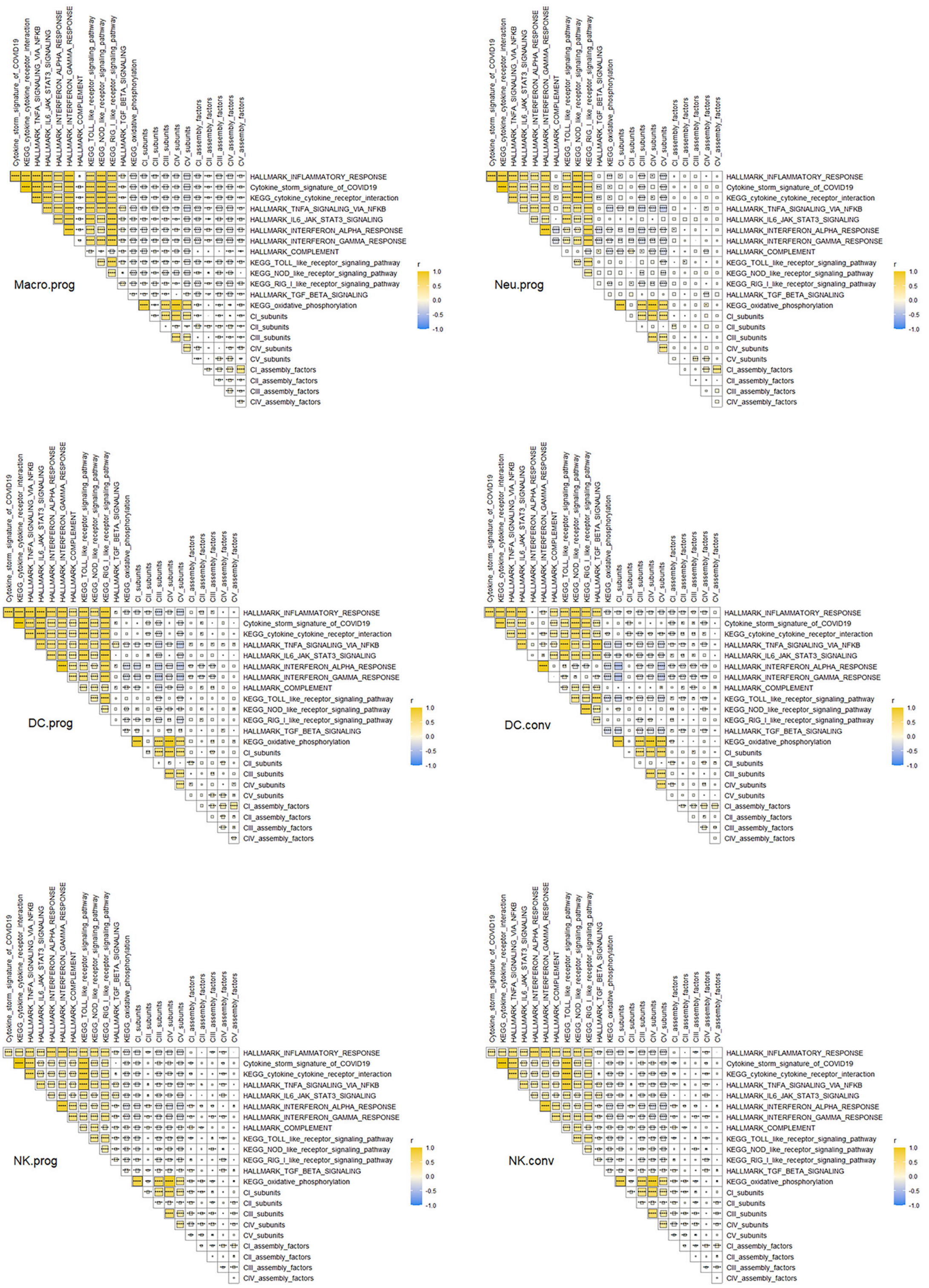

Supplementary Figure 7.

Correlations between mitochondrial OXPHOS subunits (including five core subunits and five assembly factors) and 12 immune pathways/signatures in Macro, DC, and NK from male severe/critical patients (prog or conv) of age groups 3, 5, 6, and 7.

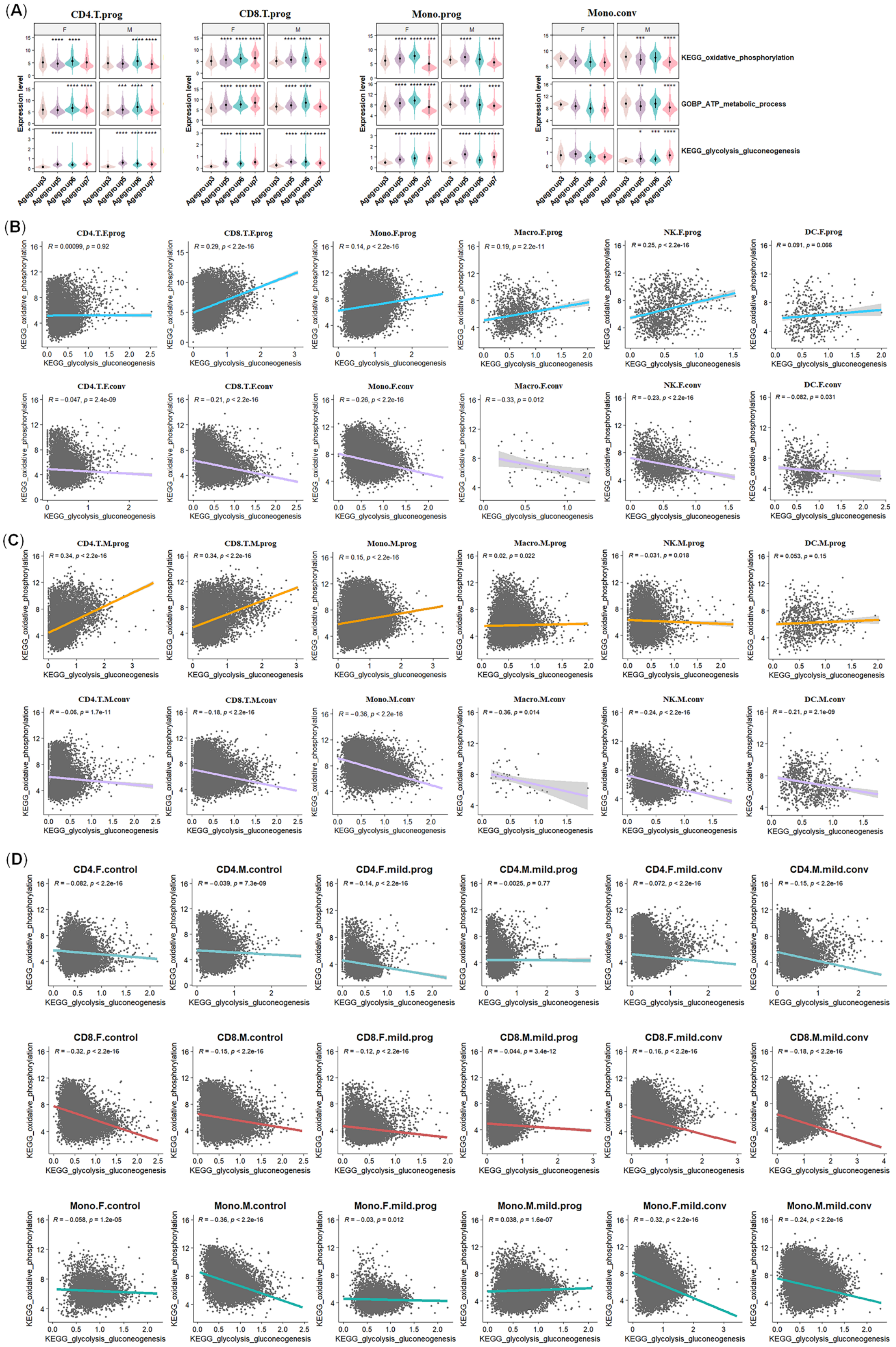

Supplementary Figure 8.

The expression dynamics of OXPHOS and glycolysis in several immune cell types from severe/critical patients in prog or conv stages. (**A**) Differential expression profiles of KEGG

oxidative phosphorylation, GOBP ATP metabolic process, and KEGG glycolysis gluconeogenesis in CD4.T cells, CD8.T cells and Mono between both genders from severe/critical patients (prog or conv). Age group 3 was set as control for statistical analysis. *p < 0.05, **p < 0.01, ***p < 0.005, ****p < 0.001, one-sided Wilcoxon rank-sum test. Mean ± SD labeled. The converse correlation of KEGG oxidative phosphorylation and KEGG glycolysis gluconeogenesis in 6 types of immune cells from female (**B**) or male patients (**C**) during disease prog and conv. (**D**) The correlation of KEGG oxidative phosphorylation with KEGG glycolysis gluconeogenesis in CD4.T (upper panels), CD8.T (middle panels) and Mono (lower panels) from normal control persons and mild/moderate COVID-19 patients (prog or conv).

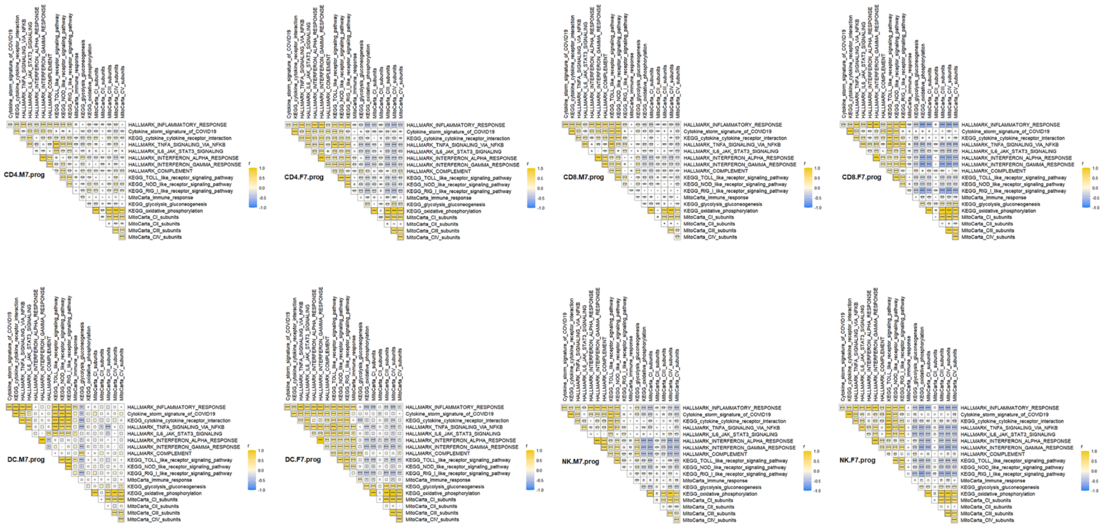

Supplementary Figure 9.

The relative dynamic expression profiles of immunity, KEGG glycolysis gluconeogenesis, and OXPHOS in CD4.T, CD8.T, Mono and Macro between both genders in age group 6 from severe/critical COVID-19 patients (prog).

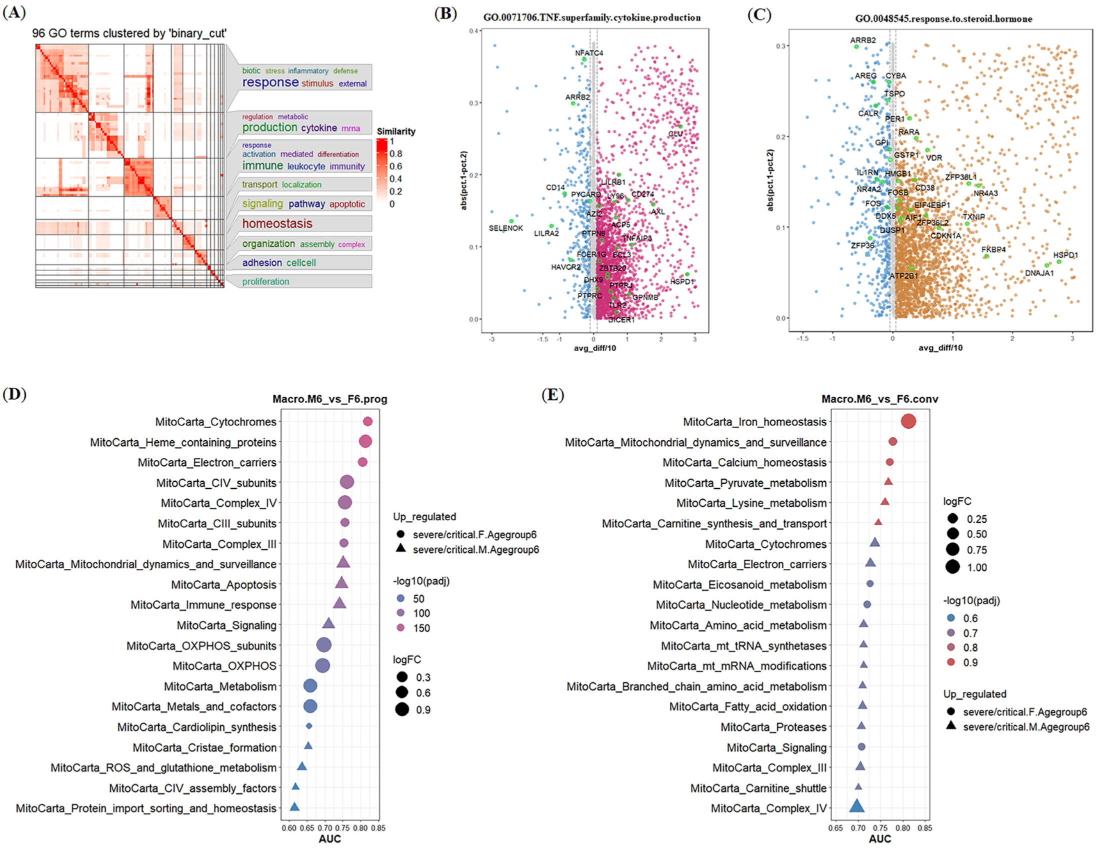

Supplementary Figure 10.

GOBP pathway enrichment analysis and the changes of mitochondrial activities between Macro.M6.prog and Macro.F6.prog. (**A**) Word-cloud annotation heatmap of the enriched GOBP pathways in DEGs between Macro.M6.prog and Macro.F6.prog. (**B**) The enriched DEGs in TNF superfamily cytokine production pathway between Macro.M6.prog and Macro.F6.prog. (**C**) The enriched DEGs in response to steroid hormone pathway between Macro.M6.prog and Macro.F6.prog. The top 20 changed MitoCarta pathways according to the ranks of AUC values in between Macro.M6.prog and Macro.F6.prog (**D**) and in between Macro.M6.conv and Macro.F6.conv (**E**).

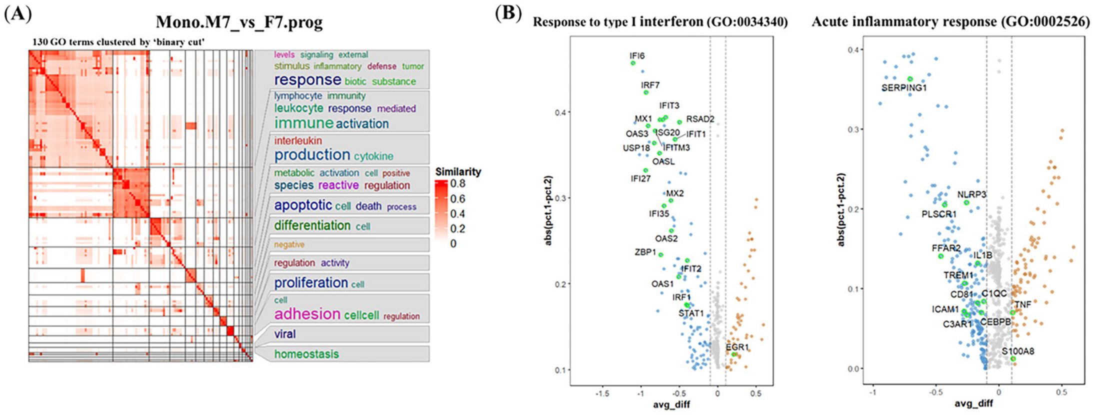

Supplementary Figure 11.

Pathway enrichment of DEGs between both genders in Mono from aged male severe/critical COVID-19 patients (≥70 years old) during disease prog. (**A**) Word-cloud annotation heatmap of the enriched GOBP pathways in differentially expressed genes between Mono.M7.prog and Mono.F7.prog. (**B**) The differentially expressed genes enriched in 2 GOBP pathways.

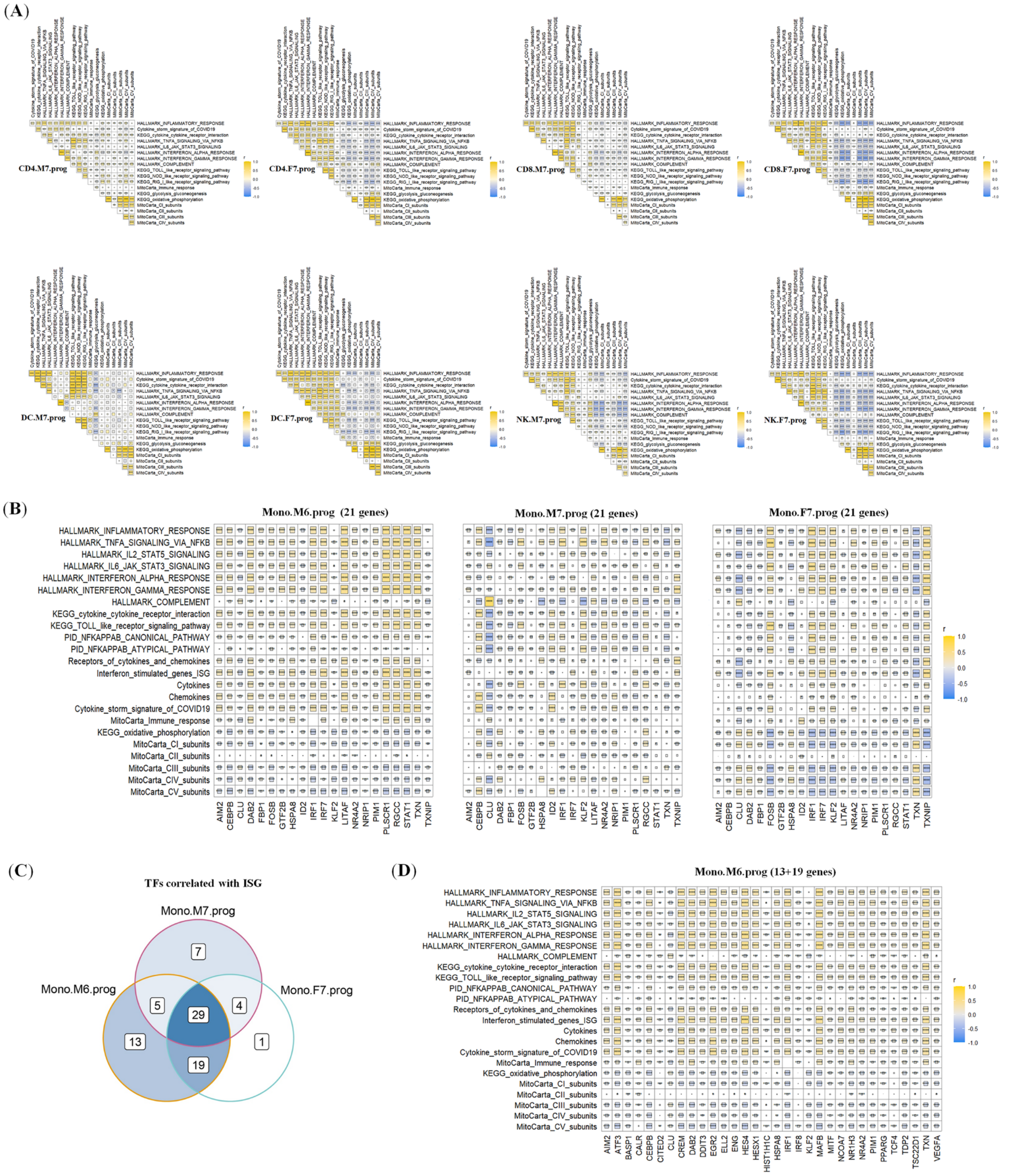

Supplementary Figure 12.

The disordered transcriptional regulation between both genders in Mono from aged male severe/critical COVID-19 patients (≥70 years old) during disease prog. (**A**) The correlations of immunity with OXPHOS in CD4.T, CD8.T, DC and NK cells between both genders in age group 7 from severe/critical COVID-19 patients (prog). (**B**) The correlation of the 23 gene

sets/pathways/signatures with the 21 TFs in Mono.M6.prog, Mono.M7.prog and Mono.F7.prog. The 21 TFs were intersected exclusively by Mono.M6.prog and Mono.F7.prog in Figure 6D. (**C**) The intersection of TFs correlated with the interferon stimulated genes ISG in Mono.M6.prog, Mono.M7.prog, and Mono.F7.prog. (**D**) The correlation of the (13 + 19) TFs in (**C**) with the 23 gene sets/pathways/signatures in Mono.M6.prog.

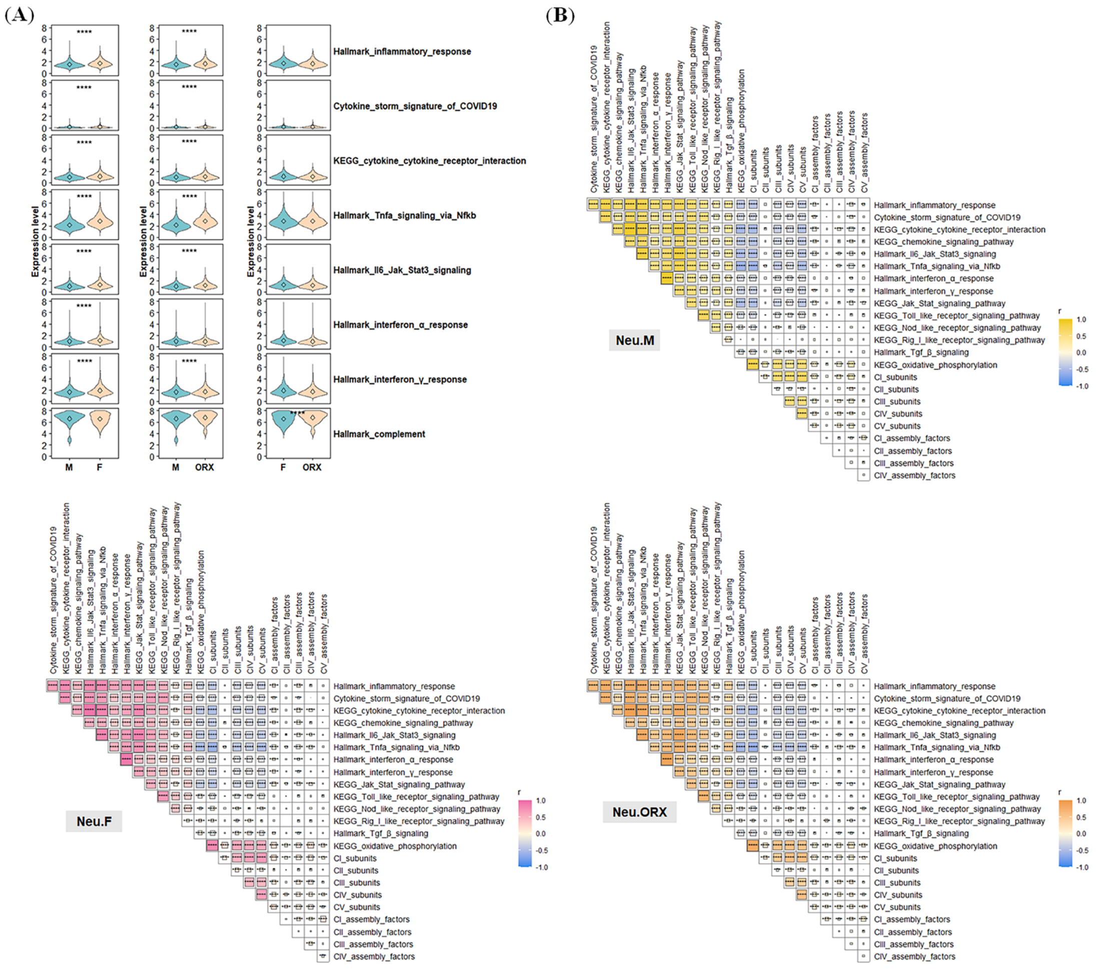

Supplementary Figure 13.

The dynamic changes of immune activities and the correlation of immunity with OXPHOS in mature Neu (bone marrows) from normal male, castrated male or normal female mice. **(A)** Pairwise comparisons of immune activities shown by the expression levels of eight pathways/gene sets/signatures in mature Neu from female mice, male mice, and castrated mice (ORX). Wilcoxon rank-sum test (alternative = "less") was performed. *p < 0.05, **p < 0.01, ***p < 0.005, ****p< 0.001. **(B)** Correlations between mitochondrial OXPHOS subunits (including five core subunits and five assembly factors) and 12 immune pathways in neutrophils from adult male mice, adult female mice, and castrated mice.

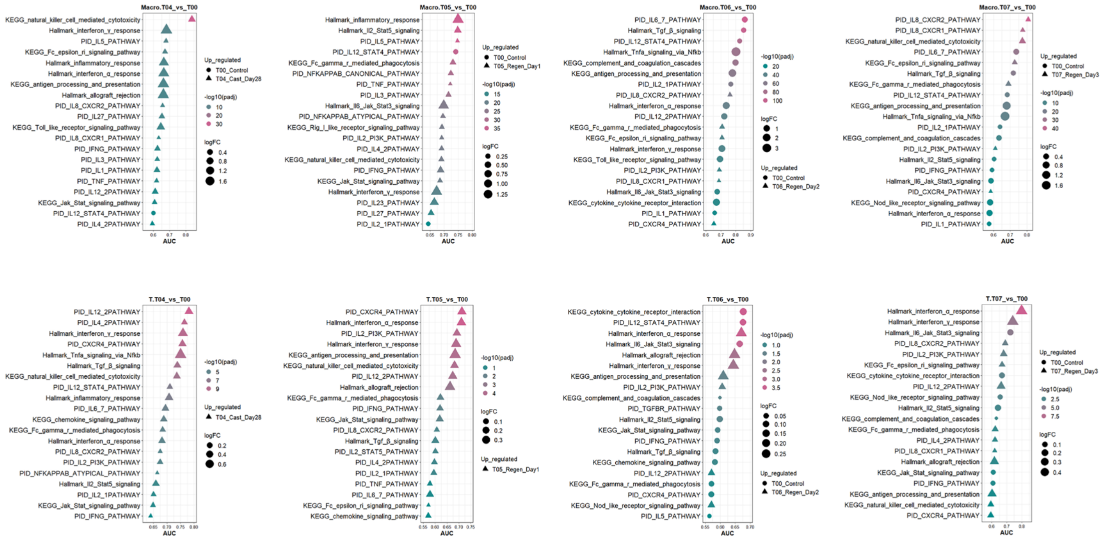

Supplementary Figure 14.

The dynamic changes of immune activities during castration and regeneration in prostate Macro (Upper panels) and T cells (lower panels).

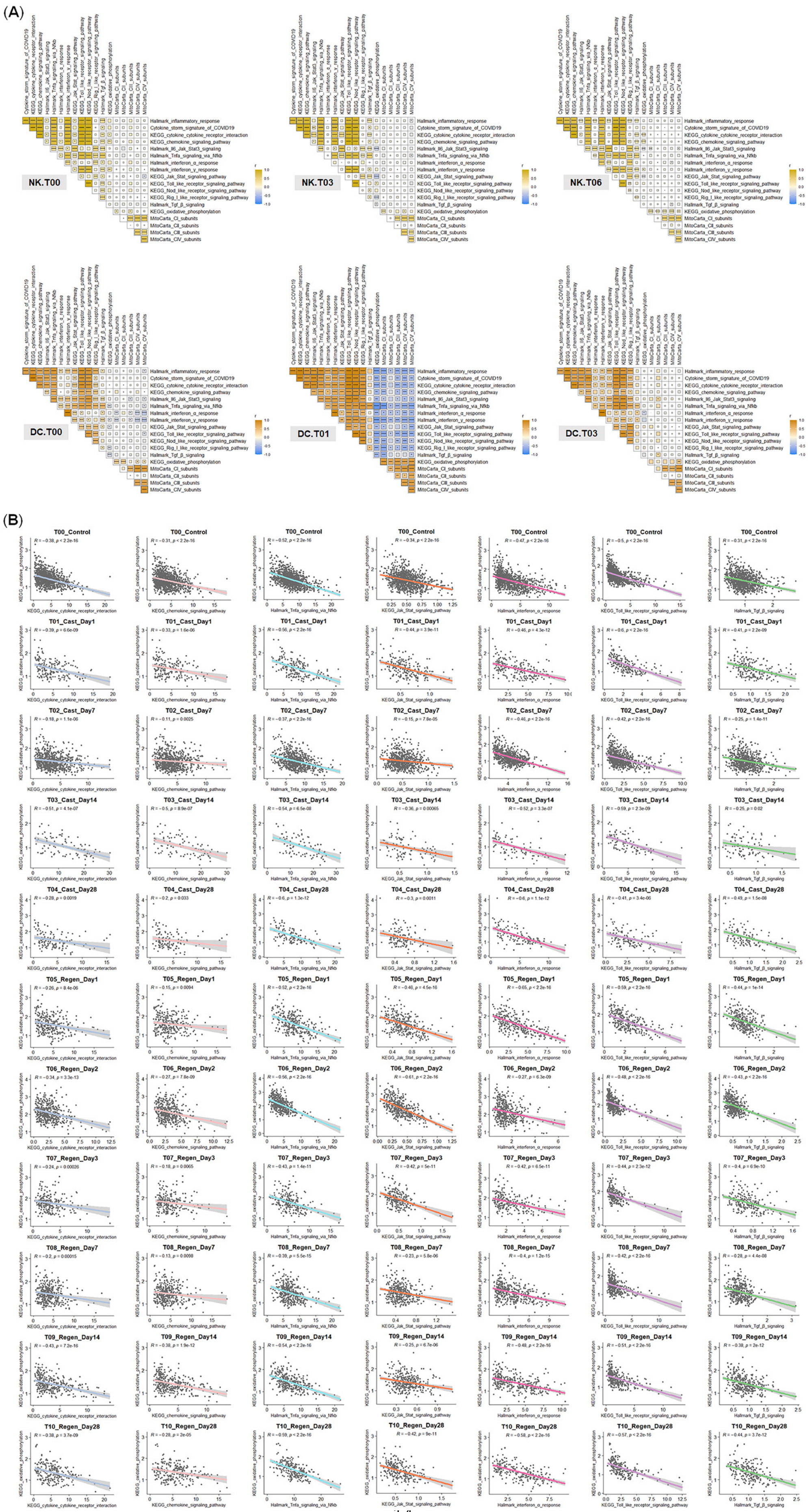
(A)
NK.T00
NK.T03
NK.T06
DC.T00
DC.T01
DC.T03
(B)
T00_Control
T01_Cast_Day1
T02_Cast_Day7
T03_Cast_Day14
T04_Cast_Day28
T05_Regen_Day1
T06_Regen_Day2
T07_Regen_Day3
T08_Regen_Day7
T09_Regen_Day14
T10_Regen_Day28

Supplementary Figure 15.

The correlation of OXPHOS with immunity in prostate immune cells from normal control, castrated and regenerated mice. (**A**) the correlation of the subunits of mitochondrial OXPHOS, KEGG oxidative phosphorylation and 12 immune pathways in NK and DC cells during castration and regeneration (**B**) Negative correlations between seven immune pathways/gene sets/signatures and KEGG oxidative phosphorylation in macrophages from intact control prostates, castration prostates, and regeneration prostates.
